# The Relationship Between Obesity, Bariatric Surgery, and Infertility: A Systematic Review

**DOI:** 10.3390/life15050758

**Published:** 2025-05-09

**Authors:** Charalampos Voros, Antonia Varthaliti, Kyriakos Bananis, Despoina Mavrogianni, Diamantis Athanasiou, Antonia Athanasiou, Aikaterini Athanasiou, Anthi-Maria Papahliou, Constantinos G. Zografos, Panagiota Kondili, Menelaos Darlas, Ioannis Papapanagiotou, Maria Anastasia Daskalaki, Marianna Theodora, Panagiotis Antsaklis, Georgios Daskalakis, Dimitrios Loutradis

**Affiliations:** 1 1st Department of Obstetrics and Gynecology, ‘Alexandra’ General Hospital, National and Kapodistrian University of Athens, 80 VasilissisSofias Avenue, 11528 Athens, Greece; antonia.varthaliti@hotmail.com (A.V.); depy.mavrogianni@yahoo.com (D.M.); anthipapahliou@gmail.com (A.-M.P.); giotakondyli27@gmail.com (P.K.); mdarlas2110@gmail.com (M.D.); md181341@students.euc.ac.cy (M.A.D.); martheodr@gmail.com (M.T.); panosant@gmail.com (P.A.); gdaskalakis@yahoo.com (G.D.); 2 King’s College Hospitals NHS Foundation Trust, London SE5 9RS, UK; kyriakos.bananis@nhs.net; 3 IVF Athens Reproduction Center V. Athanasiou, 15123 Maroussi, Greece; diamathan16@gmail.com (D.A.); antoathan16@gmail.com (A.A.); diamathan17@gmail.com (A.A.); 4 2nd Surgical Department, General Hospital of Athens “LAIKO”, 11527 Athens, Greece; koszogra92@hotmail.com; 5 Athens Medical School, National and Kapodistrian University of Athens, 15772 Athens, Greece; gpapamd@hotmail.com (I.P.); loutradi@otenet.gr (D.L.); 6 Fertility Institute-Assisted Reproduction Unit, Paster 15, 11528 Athens, Greece

**Keywords:** obesity, infertility, bariatric surgery, reproductive health, ovulatory dysfunction, pregnancy outcomes, metabolic syndrome, polycystic ovarian syndrome (PCOS)

## Abstract

Background: Obesity is a complicated, chronic condition that has a major impact on reproductive health, leading to infertility, anovulation, and poor pregnancy outcomes. It alters the hypothalamic–pituitary–ovarian (HPO) axis, promotes insulin resistance, and causes persistent low-grade inflammation, all of which result in hormonal abnormalities that compromise normal ovarian function. Because standard weight loss procedures frequently fail to provide significant and long-term reproductive benefits, bariatric surgery is becoming increasingly popular as a therapeutic option for obese women trying to conceive. However, continuous research is being conducted to determine the degree of its advantages and potential hazards to fertility and pregnancy outcomes. Methods: This systematic review was conducted in accordance with the Preferred Reporting Items for Systematic Reviews and Meta-Analyses (PRISMA) standards and entered into the PROSPERO database. Comprehensive searches in the PubMed, Scopus, and Web of Science databases turned up relevant studies. Studies that examined the effects of bariatric surgery on female fertility, ovulatory function, pregnancy rates, and neonatal outcomes were considered. Methodological quality and risk of bias were evaluated using the Newcastle–Ottawa Scale (NOS) for observational studies and the Cochrane Risk of Bias Tool for randomized controlled trials. Results: This review comprised 34 studies. More than 75% of the studies analyzed showed improvements in ovulatory function, monthly regularity, or spontaneous pregnancy after bariatric surgery. Post-surgical pregnancies are related to a lower incidence of gestational diabetes, hypertension, and macrosomia. However, several studies raised concerns about nutritional inadequacies and the possibility of small-for-gestational-age newborns, particularly following Roux-en-Y gastric bypass. Studies suggest delaying conception for 12 to 18 months after surgery to reduce nutritional hazards and improve pregnancy outcomes. Variability in study design, follow-up duration, and surgical methods reduces the generalizability of findings, emphasizing the importance of uniform research protocols. Conclusions: Bariatric surgery is a highly effective treatment for increasing fertility and pregnancy outcomes in obese women, particularly those with PCOS. However, rigorous preconception planning, postoperative nutritional monitoring, and multidisciplinary follow-up are required to reduce the related hazards. Future research should concentrate on long-term reproductive outcomes, standardizing fertility assessment criteria, and improving clinical guidelines for managing post-bariatric pregnancies. These findings support the incorporation of bariatric surgery into fertility treatment regimens for obese women, and they may shape future revisions to clinical guidelines on reproductive care following weight loss surgery.

## 1. Introduction

Obesity is a chronic and complicated condition that has reached epidemic proportions globally, with serious consequences for general health, including reproductive function. Obesity, defined by a BMI of 30 kg/m^2^ or greater, is a disorder that causes metabolic, hormonal, and inflammatory abnormalities in addition to excess body weight [1]. These changes have far-reaching effects on various physiological systems, including the cardiovascular, endocrine, and reproductive systems. Among the many difficulties associated with obesity, the impacts on female fertility and pregnancy outcomes have received a lot of attention since they affect both mother and fetal health [2]. Obese women are more likely to experience anovulation, menstrual abnormalities, infertility, and pregnancy difficulties, and obesity-related infertility is increasingly recognized as a serious public health issue. Given the expanding incidence of obesity, its influence on reproductive health has become a burgeoning subject of study, necessitating the urgent need for effective therapies that can restore fertility in afflicted women [3].

Infertility is defined as the failure to conceive after 12 months of frequent, unprotected intercourse, and it affects around 15% of all couples worldwide. While a variety of variables can contribute to infertility, including genetic illnesses and anatomical anomalies, as well as environmental and lifestyle impacts, obesity is one of the most important modifiable risk factors [4]. Obese women are significantly more likely to experience hormonal imbalances, ovulatory dysfunction, and subfertility, all of which lead to a lower chance of conceiving. Obesity has been shown in research to have a detrimental influence on menstrual cycle regularity, ovarian follicular growth, and the uterus’s ability to support implantation, resulting in decreased conception rates, an increased chance of miscarriage, and unfavorable pregnancy outcomes [5]. Obesity impairs female fertility through a variety of processes, including metabolic changes such as insulin resistance, hormone dysregulation, and chronic low-grade inflammation, all of which impair normal reproductive function [6].

Obesity causes infertility primarily through hormonal imbalance, namely in the hypothalamic–pituitary–ovarian (HPO) axis, which affects reproductive function. The HPO axis regulates the release of reproductive hormones such as GnRH, LH, FSH, estrogen, and progesterone. These hormones operate together to promote normal ovulation and prepare the uterine lining for implantation [7]. However, in obese women, extra adipose tissue creates high amounts of estrogen, which disrupts the HPO axis’s regular feedback processes. As a result, GnRH production becomes dysregulated, causing altered LH and FSH secretion patterns, resulting in irregular ovulation or anovulation. Excess estrogen from adipose tissue inhibits FSH production, inhibiting the formation and maturity of ovarian follicles required for ovulation [8].

Insulin resistance is another key cause of infertility in obese women, a disease in which the body’s cells become less receptive to insulin, resulting in hyperinsulinemia (high circulating insulin) [9]. Insulin resistance is a defining characteristic of metabolic syndrome and polycystic ovarian syndrome (PCOS), both of which are frequent among obese people. In the ovaries, insulin works in tandem with LH to promote androgen synthesis, resulting in higher levels of testosterone and other androgens [10]. This hyperandrogenic condition is typical of PCOS, in which high androgen levels lead to follicular stoppage, lack of ovulation, and monthly abnormalities. Furthermore, high insulin levels inhibit hepatic synthesis of sex hormone-binding globulin (SHBG), resulting in elevated levels of circulating free testosterone, exacerbating ovulatory failure [11]. Chronic anovulation in obese women with PCOS lowers their odds of conceiving, making it one of the most common reasons for infertility in this group [12].

Beyond hormonal abnormalities, obesity causes persistent low-grade inflammation, which further compromises reproductive function. Adipose tissue not only stores fat but also secretes inflammatory cytokines such as TNF-α, IL-6, and CRP [13]. These inflammatory mediators have been linked to decreased ovarian function, impaired follicular growth, and disrupted endometrial receptivity. Furthermore, persistent inflammation is linked to oxidative stress, which can harm oocytes (eggs), diminish mitochondrial activity in follicles, and hinder embryo implantation. Studies have also shown that heightened inflammatory markers in obese women are associated with higher rates of implantation failure, early pregnancy loss, and poor embryo quality, complicating reproductive results [14].

Given the substantial impact of obesity on reproductive health, weight loss strategies have been intensively researched as prospective fertility-restoration treatments. While lifestyle improvements, such as dietary changes and increased physical activity, might be beneficial for some people, many women with severe obesity fail to achieve and sustain considerable weight reduction using traditional techniques alone [2]. This has sparked renewed interest in bariatric surgery as a treatment option for obese women seeking to improve their fertility. Bariatric surgery is now the most effective long-term therapy for extreme obesity, resulting in significant and persistent weight loss as well as metabolic changes that directly impact reproductive health [15].

A variety of bariatric surgeries, including Roux-en-Y gastric bypass (RYGB), sleeve gastrectomy (SG), and adjustable gastric banding (AGB), have been demonstrated to enhance reproductive results in previously infertile women [16]. These surgical treatments result in significant weight loss, improved insulin sensitivity, and normalization of reproductive hormone levels, all of which contribute to restored ovulatory function and enhanced spontaneous conception rates [17]. Many women who were previously anovulatory owing to obesity or PCOS regain normal menstrual cycles and ovulation within months of surgery, greatly enhancing their chances of spontaneous pregnancy [18]. Furthermore, bariatric surgery has been linked to fewer miscarriages and better pregnancy outcomes, notably decreased risks of gestational diabetes, preeclampsia, and preterm delivery compared to obese women who conceive without weight loss assistance [19].

Despite these advantages, post-bariatric pregnancy necessitates close supervision owing to possible hazards. Women who conceive too soon after surgery are at risk of nutritional deficiencies, notably in iron, vitamin B12, folate, and vitamin D, all of which are required for embryonic growth [20]. Furthermore, studies show that pregnancies after bariatric surgery have a higher risk of having small-for-gestational-age (SGA) newborns, which might be attributable to changes in maternal nutrition absorption and metabolism. To reduce these risks, most medical standards recommend waiting at least 12 to 18 months following bariatric surgery before attempting to conceive, giving the body time to settle and adapt to postoperative changes [21].

As more women resort to surgical weight loss for fertility restoration, it is critical to establish comprehensive prenatal care techniques that maximize pregnancy outcomes while limiting possible dangers. The expanding collection of research demonstrating the beneficial effects of bariatric surgery on fertility emphasizes the need to manage obesity as part of reproductive treatment plans [17]. However, further study is needed to assess long-term reproductive results, enhance nutritional treatment, and better understand how maternal metabolic alterations affect fetal development.

This systematic review seeks to give a complete examination of the association between obesity, infertility, and bariatric surgery, examining how various surgical treatments affect fertility, pregnancy outcomes, and newborn health. By analyzing current clinical data and patient outcomes, this review will highlight the benefits and potential risks of bariatric surgery in reproductive-aged women, ultimately providing a valuable framework for future research and clinical management strategies in obesity-related infertility.

While previous studies examined several aspects of reproductive function in women undergoing bariatric surgery, many focused only on PCOS or fertility outcomes. The current review tries to combine the most recent studies on a variety of reproductive outcomes—including menstrual control, ovulatory recovery, spontaneous conception, pregnancy course, and newborn health—into a unified clinical paradigm. Our purpose is to provide clinicians with a realistic, up-to-date evidence synthesis to aid in interprofessional decision-making and patient counseling. Understanding these processes is critical for determining how bariatric surgery can correct obesity-related reproductive dysfunction. This review will look at the clinical evidence for such benefits, specifically if metabolic, hormonal, and structural changes following surgery lead to better fertility and pregnancy outcomes.

Recent systematic reviews and meta-analyses support the idea that bariatric surgery improves reproductive outcomes. For example, Al Qurashi et al. (2022) found improvements in hormonal profiles and sexual function in both men and women, whereas Makhsosi et al. (2024) reported a 67% post-surgical conception rate in infertile obese women. However, these studies only provided limited information about neonatal outcomes, conception timing, and maternal nutritional hazards. The current evaluation builds on previous studies by providing a more detailed examination of these factors, stratified by population characteristics and bariatric treatment type.

## 2. Materials and Methods

This systematic review followed the Preferred Reporting Items for Systematic Reviews and Meta-Analyses (PRISMA) standards and was prospectively registered in the PROSPERO database (CRD420250654770). The included studies underwent a comprehensive quality evaluation to determine methodological validity, danger of bias, and general dependability. Given the diversity of research types in the available literature, two well-established evaluation techniques were used: the Newcastle–Ottawa Scale (NOS) for observational studies and the Cochrane Risk of Bias Tool for randomized controlled trials. These instruments made it possible to evaluate evidence quality in an organized and consistent manner.

### 2.1. Quality Assessment

The Newcastle–Ottawa Scale (NOS) was used to evaluate observational research, which constituted the vast majority of the included studies. This scale examines the three key domains:

- Selection of research participants, including representativeness of the exposed group, selection of the non-exposed population, and exposure assessment.
- Study groups should be comparable, with research controlling for confounding variables like as age, BMI, comorbidities, or pre-existing infertility issues.
- Outcome evaluation, including adequate follow-up, clarity of outcome definitions, and possible loss due to follow-up biases.

A total NOS score (0–9) was used to categorize studies based on their methodological strength and risk of bias.

- Low risk of bias (7–9 points): Studies in this category were deemed methodologically sound, with clearly specified participant selection, extensive result evaluation, and adequate comparison procedures. These studies demonstrated substantial evidence for a link between bariatric surgery and reproductive results, with few complicating variables.
- Modest risk of bias (5–6 points): This group includes studies with modest constraints, such as small sample numbers, retrospective design, or minor methodological problems. While these studies nevertheless offered useful information, their findings were susceptible to potential biases or confounding variables that might have impacted outcomes.
- High risk of bias (0–4 points): Studies in this category have severe methodological flaws, such as selection bias, a lack of control groups, short follow-up periods, or insufficient data reporting. Their findings should be viewed with caution since there is a risk of significant bias influencing the stated results.

The Cochrane Risk of Bias Tool was used in randomized controlled trials (RCTs) to assess randomization, allocation concealment, blinding, data completeness, and selective outcome reporting. The risk of bias was rated as low, moderate, or high depending on how well certain methodological concerns were handled.

Each included study was evaluated based on numerous key methodological characteristics, including:

- Study design (prospective, retrospective, or randomized controlled trial), with prospective and RCT studies being regarded as better quality owing to their capacity to account for variables.
- Larger samples were preferred because they provided more statistical power and generalizability.
- Selection bias was evaluated based on how participants were recruited, whether rigorous inclusion criteria were followed, and if adequate control groups were employed.
- Outcome evaluation ensured that fertility, pregnancy, and neonatal outcomes were well-defined and regularly measured.
- Follow-up time, as studies with longer follow-up durations gave more information about long-term reproductive results following bariatric surgery.
- Risk of bias, which took into account concerns including recollection bias, inadequate data, loss to follow-up, and potential conflicts of interest.

The results of this assessment demonstrated substantial diversity in research quality throughout the collected literature. Studies with a NOS score of 7–9 were classified as high-quality because they used solid methodological designs, robust participant selection methods, and detailed outcome evaluations. These trials were less prone to bias and offered trustworthy information about the effects of bariatric surgery on reproductive outcomes. High-quality studies include Gosman et al., 2010, Sheiner et al., 2011, Neto et al., 2012, and Karadag et al., 2020, all with large sample numbers, extensive outcome reporting, and long follow-up periods.

Studies scoring 5–6 on the NOS scale were deemed intermediate quality and had some methodological constraints, such as small sample numbers, retrospective design, or missing confounder corrections. These studies nonetheless provided useful insights, but they should be evaluated with caution owing to possible biases. Eid et al., 2014; Legro et al., 2012; and Milone et al., 2017 are examples of studies that generated useful fertility data but lacked long-term follow-up or big enough cohorts to make conclusive conclusions.

Studies having a NOS score of 0–4 were classified as high-risk, owing to inadequate research design, insufficient data reporting, or significant methodological errors. Many of these studies suffered from selection bias, insufficient follow-up periods, or a lack of well-matched control groups, making their conclusions less credible. High-risk studies included Bilenka et al. (1995), Rochester et al. (2009), and Musella et al. (2011), which had extremely small sample sizes, poor follow-up, and potential confounding variables that were not effectively controlled for.

This quality evaluation is critical to ensure that the systematic review’s results are supported by high-quality, methodologically sound evidence. This evaluation provides a more accurate understanding of the influence of bariatric surgery on female fertility and pregnancy outcomes by classifying studies based on their risk of bias, sample size, and follow-up time. While the majority of research found beneficial connections between bariatric surgery and increased fertility, the validity of these results is heavily influenced by the quality of the studies analyzed.

The results of this quality evaluation highlight the need for future research to prioritize well-designed prospective cohort studies and randomized controlled trials with:

- Larger, more representative sample sizes will improve statistical power.
- Long-term follow-up periods are used to investigate the long-term impact of bariatric surgery on reproductive health.
- Standardized outcome measurements include fertility, pregnancy problems, and newborn health markers.
- Improved control of confounding variables such age, BMI, metabolic health, and pre-existing reproductive diseases.

As shown in Table 1, the findings of this systematic quality assessment give a thorough review of the included studies’ strengths and weaknesses, allowing for a more trustworthy synthesis of the existing information on bariatric surgery as a reproductive intervention. This organized methodology guarantees that the findings of this systematic review are based on the best available evidence, reducing the impact of methodological biases and emphasizing opportunities for future study improvements.

For RCTs, the Cochrane Risk of Bias Tool was used to assess the randomization procedure, blinding measures, outcome data completeness, and selective reporting bias. Methodological rigor determined whether studies had a low, moderate, or high risk of bias. To guarantee consistency and eliminate subjectivity, the quality evaluation was undertaken by two independent reviewers. Cohen’s kappa coefficient (κ) was used to assess inter-rater reliability, with levels of agreement ranging from minor (0.01–0.20) to practically perfect (>0.80).

Table 1 shows the quality evaluation of the papers included in this systematic review sheds light on the reliability, validity, and generalizability of the results addressing the influence of bariatric surgery on female fertility. Given the complexities of obesity-related infertility and the challenges of performing long-term clinical trials in this group, study methodological quality varied greatly. This analysis will focus on important features of study design, sample size, bias concerns, follow-up duration, and overall dependability, offering a nuanced view of the strengths and limits of the current body of research.

The research design plays an important role in determining the credibility of the findings in this systematic review. The bulk of the included studies (23 out of 33) were retrospective, which means that data were gathered from previous medical records or databases rather than controlled, prospective research. Retrospective studies are inherently more biased, especially owing to probable recollection bias, missing data, and errors in medical record-keeping. These constraints can have an influence on the reliability of reported outcomes such as menstrual cycle regularity, spontaneous ovulation, pregnancy rates, and newborn problems.

However, few retrospective investigations stood out because of their methodological rigor. Sheiner et al., 2011 and Gosman et al., 2010, both with large sample sizes (489 and 1538 participants, respectively), reported well-documented pregnancy outcomes, which strengthened their conclusions. Long-term follow-up studies, such as Marceau et al., 2004 and Neto et al., 2012, provided vital insights on persistent reproductive benefits following bariatric surgery. Despite the inherent flaws of retrospective designs, large sample numbers and thorough follow-up increased the trustworthiness of their results. In contrast, prospective research (e.g., Legro et al., 2012, Nilsson-Condori et al., 2018, and Sahab Al Kabbi et al., 2018) allowed for more controlled data collecting and decreased recall bias. These studies prospectively followed women after bariatric surgery and evaluated their reproductive results using established criteria. Prospective studies are more credible because they allow researchers to control confounding variables and examine reproductive results consistently across time. The highest quality study among all the included studies was Samarasinghe et al.’s 2024 randomized controlled trial (RCT). This study directly compared bariatric surgery to medicinal weight loss therapies in women with PCOS, using randomization and blinding to eliminate bias. The randomized controlled trial design is the gold standard in clinical research because it reduces confounding variables and provides the most reliable assessment of treatment effects. With its solid methodology and precise fertility outcome indicators, this study offered the most conclusive data confirming the importance of bariatric surgery in restoring reproductive function.

The included studies’ sample sizes ranged from as few as 9 individuals (Bilenka et al., 1995; Rochester et al., 2009) to over 1500 participants (Gosman et al., 2010). In general, bigger sample sizes produce more trustworthy and statistically significant results by reducing the effect of random variation.

- Large sample size studies (e.g., Gosman et al., 2010, Sheiner et al., 2011, Neto et al., 2012) were evaluated as high-quality because their findings were more generalizable to the larger population of women undergoing bariatric surgery for infertility therapy.
- Studies with a smaller sample size (e.g., Bilenka et al., 1995, Rochester et al., 2009, and Musella et al., 2011) had a higher risk of bias and were ranked worse in quality due to less statistical power and generalizability.
- Studies on PCOS populations (e.g., Eid et al., 2005, Jamal et al., 2012, Ezzat et al., 2021) had moderate quality ratings due to limited sample sizes, but they gave particular insights into how bariatric surgery impacts fertility in PCOS individuals.

In general, research with higher sample numbers and clearly specified fertility outcomes was more trustworthy, but studies with lower sample sizes should be regarded with care.

Selection bias was another aspect influencing research quality. Some research used rigorous inclusion and exclusion criteria, which may restrict the application of the findings to larger groups.

- High-quality research (e.g., Gosman et al., 2010, Sheiner et al., 2011, Karadag et al., 2020) enrolled a varied variety of individuals with various levels of obesity and reproductive health concerns, making their findings more generalizable to the general population.
- Studies that focused primarily on PCOS patients (e.g., Eid et al., 2005, Jamal et al., 2012, and Ezzat et al., 2021) showed lower external validity since their findings only applied to a subset of obese women experiencing infertility.
- Studies with relatively small sample sizes (e.g., Bilenka et al., 1995; Rochester et al., 2009) were more prone to selection bias, as their findings may not be typical of the larger population.

Overall, research with varied participant demographics and broad inclusion criteria was regarded as higher quality due to increased external validity.

One of the most important components of research quality was the length of the follow-up period, as fertility and pregnancy results might take years to appear after surgery.

- Long-term follow-up studies (e.g., Marceau et al., 2004, Neto et al., 2012, Karadag et al., 2020) have offered important insights into the long-term durability of fertility improvements and pregnancy outcomes following bariatric surgery.
- Studies with short follow-up periods (e.g., Rochester et al., 2009, Sahab Al Kabbi et al., 2018, and Jamal et al., 2012) received lower ratings due to a lack of data on long-term reproductive outcomes.

The best-designed studies included long-term follow-up and measured not just pregnancy rates but also newborn health outcomes, allowing for a more thorough assessment of bariatric surgery’s impact on reproductive health.

Taking these variables into account, the studies with the best ratings in this systematic review used a prospective or randomized controlled design, such as those conducted by Samarasinghe et al. (2024) and Menke et al. (2019). These studies were deemed of better quality because they were able to control confounding factors and reduce bias, resulting in more trustworthy information on the influence of bariatric surgery on reproductive outcomes. Furthermore, studies with higher sample sizes, such as those by Gosman et al. (2010) and Sheiner et al. (2011), received good marks since they provided more statistically robust results that could be generalized to larger populations. A prolonged follow-up time also led to greater research quality, as seen by the work of Neto et al. (2012) and Karadag et al. (2020), allowing for a more thorough assessment of the long-term consequences of bariatric surgery on reproductive health. Furthermore, studies that examined pregnancy and neonatal outcomes in depth, such as those by Snoek et al. (2023) and Joly et al. (2024), gave important insights into the mother and fetal consequences of post-surgical pregnancies.

In contrast, the lowest rated studies had small sample numbers and short follow-ups, decreasing the findings’ reliability and generalizability. For example, the investigations by Bilenka et al. (1995) and Rochester et al. (2009) had a limited number of participants, reducing statistical power and making results less definitive. Furthermore, studies with a high risk of recall bias, particularly those with retrospective designs, received poorer quality ratings due to the inherent limits of depending on previous medical data and patient recollection. Another aspect leading to inferior research quality was the implementation of tight participant inclusion criteria, which reduced the findings’ application to a larger population. Some studies, for example, focused solely on women with polycystic ovarian syndrome (PCOS), rendering their findings less applicable to obese women experiencing infertility from other reasons. Overall, while most studies found a link between bariatric surgery and better reproductive results, the heterogeneity in study design, sample size, and follow-up duration emphasizes the need for future research using more rigorous procedures to bolster the evidence foundation.

Given the complexities of fertility-related outcomes, various confounding variables such as age, BMI, polycystic ovarian syndrome (PCOS), insulin resistance, and ART treatment were carefully considered. Studies that included these factors in their analysis were evaluated as better quality, whereas those that did not correct for major confounders were scored lower owing to potential bias. Confounding variables were not well accounted for; therefore, results were taken with caution, and sensitivity analyses were completed to examine their influence on findings.

**Figure 1:**
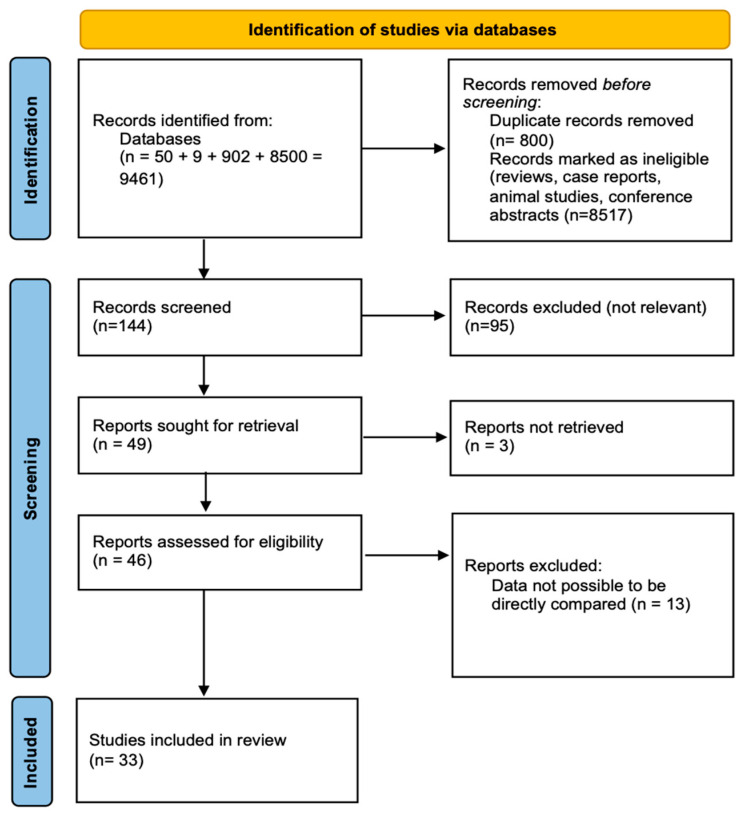
PRISMA Flow Diagram of Study Selection Process.

To establish a complete and systematic approach to identifying relevant research, the Preferred Reporting Items for Systematic Reviews and Meta-Analyses (PRISMA) 2020 framework was used. This method made the selection of papers for this systematic review more apparent. Figure 1 depicts the PRISMA flow diagram, which shows the steps involved in study identification, screening, eligibility evaluation, and final inclusion. The graphic depicts how studies were retrieved from various databases, the number of records that were removed due to irrelevance or duplication, and the total number of studies included in the systematic review. Supplementary Table S1 contains the full search strings for all databases. Only items published in English were considered suitable for inclusion.

### 2.2. Eligibility Criteria

To guarantee the inclusion of relevant, high-quality studies in this systematic review, specified eligibility criteria were developed based on research design, demographic characteristics, exposure (bariatric surgery), and outcome measures. Studies were considered if they offered quantitative data on fertility outcomes in women who had undergone bariatric surgery, allowing for an evaluation of the procedure’s influence on reproductive health.

#### 2.2.1. Inclusion Criteria

Eligible studies met the following criteria:

The studies had to be observational cohort studies (prospective or retrospective), case–control studies, or randomized controlled trials (RCTs) that looked at the effects of bariatric surgery on female fertility, reproductive hormone levels, ovulatory function, menstrual cycle regularity, pregnancy rates, or neonatal outcomes. To maintain methodological rigor, studies have to provide fertility-related quantitative outcomes such as conception rates, ovulation restoration, assisted reproductive technology (ART) success rates, pregnancy problems, and neonatal variables. Only studies with a minimum follow-up duration of six months post-surgery were included, which allowed for the evaluation of either short-term reproductive changes (e.g., menstrual and hormonal improvements) or, when available, pregnancy and neonatal outcomes in studies with extended follow-up. Furthermore, studies were needed to distinguish between preoperative and postoperative fertility results in order to show a direct link between bariatric surgery and reproductive benefits.

#### 2.2.2. Exclusion Criteria

Studies were omitted if they did not satisfy the basic requirements for scientific rigor or relevance to fertility outcomes. To ensure that only original, high-quality research papers were included, we removed review articles, systematic reviews, meta-analyses, conference abstracts, case reports, and non-peer-reviewed publications. Animal studies, in vitro investigations, and research without human clinical data were also rejected. Studies that did not provide a clear distinction between preoperative and postoperative reproductive outcomes, or that did not offer quantitative fertility data, were eliminated. Furthermore, studies with high attrition rates (>30%) or insufficient follow-up data on pregnancy outcomes were deemed untrustworthy and eliminated. This evaluation also excluded studies that focused primarily on male reproductive outcomes following bariatric surgery or fertility therapies unrelated to bariatric surgery.

A meta-analysis was not performed because of significant variation in study design, population characteristics, definitions of reproductive outcomes, surgical techniques, and follow-up lengths. These technical and clinical discrepancies reduced data comparability and prevented effective statistical pooling.

Disagreements amongst reviewers during the screening or data extraction process were handled through discussion and mutual consent. When consensus could not be reached, a third senior author was consulted to make the ultimate decision.

## 3. Results

This section covers the results of a comprehensive review on the effects of bariatric surgery on female fertility and pregnancy outcomes. A total of X studies satisfied the inclusion criteria and were evaluated for fertility outcomes, pregnancy problems, and neonatal health markers. The findings first outline the features of the included studies, including research design, sample size, and kind of bariatric surgery conducted. This is followed by an examination of fertility-related enhancements, such as menstrual cycle control, ovarian function, and conception rates. Pregnancy outcomes such as gestational diabetes, hypertensive diseases, miscarriage rates, and delivery procedures are also investigated, as are neonatal health indicators such as birth weight and the prevalence of small-for-gestational-age (SGA) newborns. A quality evaluation of the included studies is used to examine methodological strengths and weaknesses that may affect the trustworthiness of the findings. Where relevant, comparisons of various bariatric operations are presented. These findings give a complete summary of bariatric surgery’s advantages and possible hazards to reproductive health.

Of the 34 studies considered, 14 were solely focused on women with PCOS, whereas 20 looked at general obese populations or mixed reproductive-age cohorts. Because of the unique pathophysiology aspects of PCOS, such as hyperandrogenism and insulin resistance, the reproductive outcomes of these subgroups are reported and interpreted individually throughout the review, where data permit.

### 3.1. Study Selection and Characteristics

The final synthesis comprised 34 studies that met the inclusion criteria. The PRISMA flow diagram (Figure 1) illustrates the selection process. These studies differed in methodology, sample size, type of bariatric surgery, and follow-up period, as described below.

The PRISMA flow diagram, in Figure 1, depicts the research selection process, including the number of records found, vetted, and included in the final systematic review. The identification phase displays the number of records collected from databases (n = 9461) and the number of duplicate and ineligible entries eliminated prior to screening (n = 9317). During the screening phase, 144 records were assessed for relevance, resulting in the elimination of 95 irrelevant studies. Following that, 49 reports were requested for full-text retrieval, with 3 not retrievable. The remaining 46 reports were evaluated during the eligibility phase, with 12 being removed owing to insufficient data comparability. The final review comprised 34 studies.

The PRISMA flow diagram outlines the methodical process used to find, filter, and select papers for inclusion in this review. This thorough selection procedure promotes transparency, repeatability, and scientific rigor, reducing bias and increasing the relevance of the included research. By breaking out the various rounds of study selection, the figure emphasizes the search strategy’s strengths and limitations, as well as the possible influence of study attrition on the final review results.

The identification phase is the initial step in the systematic review process, involving a thorough search of numerous scientific databases and sources to acquire relevant research. A total of 9461 documents were found, indicating a broad and comprehensive search approach focused on gathering all available information on the effects of bariatric surgery on female fertility and pregnancy outcomes. The enormous number of first reports emphasizes the importance of the issue and the rising corpus of research on metabolic and reproductive health linkages.

To enhance efficiency, duplicate records were eliminated prior to screening, resulting in a reduction of 800 records. Duplicates emerge when different databases index the same study, and removing them is a critical step in avoiding repeated analysis. Following that, an additional 8517 records were removed based on initial eligibility criteria, with the majority being review articles, case reports, editorials, animal studies, conference abstracts, and research unrelated to reproductive outcomes. This phase guaranteed that only primary research studies including human clinical data were chosen for further review. While removing irrelevant research helps to tighten the review focus, it also increases the risk of omitting grey literature or unpublished data, which may include useful insights. The analysis favors peer-reviewed, high-quality papers, but future research should look at broadening sources to include preprint repositories and clinical trial registries to capture ongoing research.

After deleting duplicates and ineligible records, 144 studies proceeded to the screening step. During this step, papers were evaluated according to their title and abstract content to establish their relevance to the research issue. Screening is an important step for excluding research that, while first looking relevant, does not match the precise inclusion requirements. At this point, 95 studies were deemed irrelevant. Common causes for exclusion included the following:

- Weight reduction therapies have been studied without regard for reproductive results.
- Research on male fertility after bariatric surgery.
- Studies that describe metabolic or endocrine results without specifically assessing monthly regularity, ovulation, pregnancy rates, or newborn health.
- Population-based research in which reproductive results were not divided according to bariatric surgery status.

The screening approach helps to reduce the emphasis on studies with measurable reproductive outcomes, hence boosting the systematic review’s specificity and therapeutic relevance. However, abstract-based screening has drawbacks, since some relevant studies may be eliminated owing to inadequate information in their abstracts. To reduce this risk, studies with partial abstracts were tagged as possibly relevant and preserved for full-text evaluation.

After evaluating the titles and abstracts, 49 papers moved on to the full-text assessment. This step is crucial for verifying that studies fit all inclusion criteria and that their methodology is consistent with the review’s aims. Full-text assessment enables a thorough examination of research design, demographic characteristics, intervention details, fertility results, and follow-up time.

During the full-text assessment step, three papers were unable to be obtained due to paywall restrictions, inaccessible full-text versions, or access limits in the databases examined. Despite efforts to obtain these studies through institutional access, their absence creates a possible knowledge vacuum. This restriction highlights the difficulties of systematic reviews, where limited access to public data can lead to unintended exclusions that could have influenced the overall conclusions. In addition, twelve studies were removed because their data were not comparable. These exclusions were essential to ensure the review’s methodological integrity. Some studies lacked pre- and post-surgical fertility data, making it impossible to precisely link reproductive changes to bariatric surgery. Others utilized diverse outcome definitions, including fertility measurements that differed greatly from those used in the included studies, restricting the capacity to properly combine data. Furthermore, studies with significant attrition rates (greater than 30% of subjects lost to follow-up) were removed since their trustworthiness was questioned. While excluding these studies helped to ensure analytic consistency, it also may have caused selection bias by restricting the evaluation to trials with well-specified fertility outcomes.

Following this stringent selection procedure, a total of 34 articles were included in the systematic review, laying the groundwork for the subsequent analysis. These studies satisfied all of the inclusion requirements, indicating their importance in assessing the impact of bariatric surgery on menstrual control, ovulatory function, pregnancy rates, and perinatal health outcomes. The selected studies were diverse in terms of research design, ranging from prospective and retrospective cohort studies to randomized controlled trials (RCTs). There was also a wide range of bariatric surgical procedures performed, including Roux-en-Y gastric bypass (RYGB), sleeve gastrectomy (SG), adjustable gastric banding (AGB), and biliopancreatic diversion (BPD). Furthermore, follow-up periods varied, with some studies examining short-term hormonal changes within 6 to 12 months, while others studied long-term fertility and pregnancy outcomes for beyond five years.

Although these studies give useful information on the relationship between bariatric surgery and reproductive outcomes, significant limitations remain. One of the most significant issues is the heterogeneity in fertility outcome definitions among research studies, which makes direct comparisons difficult. The lack of consistent follow-up lengths is also a problem, as reproductive improvements might occur at varying rates based on individual metabolic responses and weight reduction trajectories. Furthermore, variances in study quality, as shown by the risk-of-bias evaluation, may alter the dependability of reported findings, emphasizing the need for more standardized research procedures in this sector.

The PRISMA flow diagram depicts the methodical and transparent process for study selection, highlighting the rigor utilized in screening and eligibility evaluation. The selection of 33 studies from an initial pool of 9461 records demonstrates the review’s very selective character, which ensured that only clinically relevant, high-quality material was included. The large number of initial exclusions (9317 items eliminated before screening) emphasizes the need for precise eligibility criteria in refining literature searches. The decrease from 144 screened records to 33 included studies indicates the thorough selection procedure, which ensured that only research with well-specified fertility outcomes was studied. The removal of non-retrievable and non-comparable studies reflects the difficulties commonly faced in systematic reviews, notably in terms of acquiring complete texts and standardizing outcome measures. Despite these limitations, the systematic methodology employed in this study guarantees that the findings are grounded in methodologically solid data, reducing the possibility of bias and increasing the dependability of conclusions on the influence of bariatric surgery on female fertility.

### 3.2. Study Designs and Methodological Features

The methodological characteristics of the included studies, in Table 2, shed light on the association between bariatric surgery, fertility, and pregnancy outcomes. The analysis emphasizes the variety of study designs, participant selection criteria, types of bariatric surgeries, and fertility-related outcomes, highlighting both the benefits and limitations of the present body of data. This expanded analysis delves into significant characteristics of the included studies, considering their implications for clinical practice and future research possibilities.

The systematic review uses a range of study methods, including retrospective cohort studies, prospective cohort studies, and one randomized controlled trial (RCT). The prevalence of retrospective research emphasizes the practical and ethical difficulties in conducting longitudinal fertility trials requiring surgical procedures. While retrospective designs are important for discovering long-term trends, they are intrinsically constrained by hazards such as recollection bias, inadequate data, and variability in outcome reporting.

In contrast, prospective studies, such as those by Rochester et al. (2009), Menke et al. (2019), and Sahab Al Kabbi et al. (2018), provided more systematic data collection and increased consistency. However, smaller sample sizes were frequently used, potentially reducing statistical power. The single RCT included in this study, conducted by Samarasinghe et al. (2024), used high-quality methodology and indicated increased ovulatory function and menstrual regularity after bariatric surgery. Nonetheless, it did not evaluate long-term pregnancy or newborn outcomes and had a small sample size.

Exclusion criteria varied, but frequently included the presence of endocrine problems, hormonal contraception usage, or male factor infertility. These variances may have an impact on the comparability and generalizability of results across populations.

The included trials included a variety of bariatric operations, including Roux-en-Y gastric bypass (RYGB), sleeve gastrectomy (SG), adjustable gastric banding (LAGB), and, less commonly, biliopancreatic diversion. Each technique has unique physiological effects, dietary ramifications, and possible reproductive results. For example, RYGB was the most extensively investigated and proved to be successful for fertility restoration, but it was also linked to an increased risk of miscarriage and small-for-gestational-age (SGA) infants. SG was typically good for fertility, although the neonatal effects varied across research. LAGB was associated with lower nutritional hazards but less significant changes in reproductive outcomes. BPD, albeit less prevalent, exhibited significant reproductive advantages but needed close postoperative dietary monitoring.

The delay between surgery and conception ranged from less than 12 months to more than 10 years. Many studies, like Sheiner et al. (2011), Neto et al. (2012), and Jacamon et al. (2020), recommend deferring conception for 12 to 18 months after surgery to reduce the hazards of fast weight loss and nutritional deficits.

The studies’ inclusion criteria were various. While the majority of studies included obese women having bariatric surgery, Eid et al. (2005), Jamal et al. (2012), and Ezzat et al. (2021) concentrated on patients with polycystic ovarian syndrome (PCOS). These results point to a particular effect of bariatric surgery in restoring ovulatory function in PCOS patients. Others, such as Milone et al. (2017) and Grzegorczyk-Martin et al. (2020), focused on women who had previously used assisted reproductive technology (ART). Age limits were also frequent, with most research limited to women aged 18 to 45, while some studies, such as Nilsson-Condori et al. (2018), included older participants.

To reduce maternal and fetal risks, clinicians should take a multidisciplinary approach that includes regular nutritional monitoring, targeted supplementation (particularly iron, vitamin B12, folate, and vitamin D), and careful pregnancy planning, with conception delayed until 12–18 months postoperatively.

### 3.3. Fertility and Neonatal Outcomes

Across the included studies, bariatric surgery was consistently associated with significant improvements in ovulatory function, menstrual cycle regulation, and spontaneous conception rates. This was particularly evident in PCOS populations, with several studies, such as Eid et al. (2014) and Samarasinghe et al. (2024), reporting restored ovulatory function within six to twelve months post-surgery. Jamal et al. (2012) and Musella et al. (2012) noted spontaneous pregnancy rates ranging from 62% to 100% in women who had previously experienced infertility. Improvements in pregnancy outcomes have also been widely recognized. Common findings included lower rates of gestational diabetes and hypertension. However, certain studies, such as Cruz et al. (2019) and Goldman et al. (2016), found higher miscarriage rates, particularly in pregnancies occurring soon after surgery. Neonatal outcomes vary. Many studies have found decreased incidence of macrosomia and metabolic problems among babies. However, Jacamon et al. (2020) and Joly et al. (2024) reported higher rates of SGA newborns and neonatal intensive care unit (NICU) hospitalizations in early post-surgical pregnancies.

### 3.4. Patient Characteristics

Table 3 summarizes the demographic and clinical characteristics of the patients who participated in the studies included in this systematic review. It includes data on the number of patients, their age, preoperative and postoperative BMI, follow-up period, prevalence of polycystic ovarian syndrome (PCOS), and reasons for infertility. The data given here show the baseline obesity status of patients undergoing bariatric surgery, the degree of weight loss achieved after surgery, and the most prevalent infertility-related issues that necessitated surgical intervention.

The included research consistently shows that bariatric surgery improves many aspects of female reproductive health. Most studies found that weight loss after surgery resulted in the restoration of regular menstrual cycles, a reduction in hyperandrogenism, and the reestablishment of ovulatory function, particularly among women with PCOS. While the kind of treatment, period of follow-up, and criteria used to quantify fertility outcomes varied, a general tendency was an increase in spontaneous pregnancy after surgery, particularly within 12 to 24 months. The majority of studies showed positive pregnancy outcomes, while a subset emphasized potential nutritional inadequacies and hazards of small-for-gestational-age newborns, particularly after malabsorptive operations. Despite the heterogeneity of the evidence, the overall synthesis implies that bariatric surgery may improve fertility and pregnancy, as long as proper nutritional monitoring and follow-up are performed.

This review comprised 34 studies. Fourteen of these focused on women with polycystic ovary syndrome (PCOS), whereas the other twenty covered mixed populations or women without the condition. The most well-investigated operations were Roux-en-Y gastric bypass (RYGB) and sleeve gastrectomy (SG). Across the included trials, 27 out of 34 (or roughly 79%) found improvements in at least one fertility-related outcome, such as menstrual regulation, ovulatory function, or spontaneous conception. Nine studies looked at pregnancy-specific outcomes, and seven of them linked bariatric surgery to improved pregnancy or neonatal metrics. These findings indicate a generally upward trend, despite variations in technique, follow-up time, and population characteristics.

The demographic and clinical features of the patients in these studies give a complete picture of the effects of bariatric surgery on obesity-related infertility, reproductive health, and pregnancy outcomes. The heterogeneity in sample sizes, age distributions, pre- and post-surgical BMI, follow-up lengths, PCOS prevalence, and underlying infertility reasons highlights the challenge of assessing reproductive outcomes after bariatric surgery. The findings of this review highlight that many included studies report positive effects of bariatric surgery on fertility restoration and menstrual regulation, particularly in women with obesity-related anovulation or PCOS.

Each study included a different number of patients, ranging from small cohorts of less than 10 patients in Bilenka et al. (1995) and Rochester et al. (2009) to bigger datasets with over 1500 participants in Gosman et al. (2010) and Menke et al. (2019). Larger sample sizes provide more statistical power and more generalizable conclusions, but smaller studies may be more susceptible to selection bias and may be unable to identify minor impacts on reproductive outcomes.

The age distribution of patients is another key aspect in evaluating fertility results. The majority of patients in these trials were between the ages of 18 and 45, however, Gosman et al. (2010) and Neto et al. (2012) included patients as elderly as 78 and 62 years old, respectively. The inclusion of older patients presents a confounding variable, as age-related fertility reduction is a major factor irrespective of obesity and weight loss. Pregnancy rates in research, including elderly adults, may be lower due to decreased ovarian reserve and reproductive potential. This suggests that age should be a major consideration when counseling patients about post-bariatric surgery fertility outcomes, as younger patients may have a better chance of spontaneous conception after surgery, whereas older patients may still need assisted reproductive technologies (ART) to conceive.

One of the most striking findings across studies was a considerable drop in BMI after bariatric surgery, highlighting the importance of weight loss in enhancing reproductive function and general metabolic health. Prior to surgery, most patients had severe obesity (BMI > 40 kg/m^2^), with some studies involving patients with extreme obesity (BMI > 50 kg/m^2^), such as Jamal et al. (2012) and Neto et al. Although postoperative BMI reductions varied between trials, most patients achieved a considerable drop in body weight, typically attaining a BMI in the overweight or moderate obesity range (BMI 25–35 kg/m^2^). Some studies, such as Legro et al. (2012) and Milone et al. (2017), offered thorough longitudinal tracking of BMI changes over time, revealing that fertility improvements were frequently associated with the degree of weight reduction accomplished. However, a lower BMI alone may not be enough to predict fertility restoration. Musella et al. (2012) found that patients who became pregnant after surgery had a lower BMI (34.2 kg/m^2^) than those who remained infertile (41.5 kg/m^2^), indicating that achieving a BMI closer to the normal range may lead to higher reproductive benefits. Furthermore, the persistence of obesity in certain individuals after surgery, as reported by Sheiner et al. (2004), may suggest that variables other than BMI, such as hormonal and metabolic alterations, influence reproductive results.

The follow-up period varied greatly between studies, ranging from 65 days (Sahab Al Kabbi et al., 2018) to more than 10 years (Marceau et al., 2004; Neto et al., 2012). Longer follow-up periods give more relevant insights into long-term fertility restoration, pregnancy outcomes, and newborn health, whereas shorter trials may not capture all of the reproductive advantages of bariatric surgery. Extended follow-up studies consistently showed long-term reproductive gains and metabolic advantages. For example, Neto et al. (2012) discovered that infertility remission was maintained over time, even in individuals who gained weight. In contrast, in trials with shorter follow-up periods, such as Ezzat et al. (2021), no pregnancies were recorded within one year of surgery, emphasizing the necessity for prolonged monitoring to identify actual fertility changes. Another important aspect is the time of conception following surgery. Many studies advocate waiting at least 12–18 months before trying conception, since significant weight loss and metabolic adjustments in the first year after surgery might result in nutritional shortages and pregnancy problems. However, some studies, such as Karadag et al. (2020) and Gunakan et al. (2019), discovered that pregnancies within the first year after surgery were not always associated with poor outcomes, implying that optimal conception timing may vary depending on individual patient characteristics and nutritional status.

Of the 14 trials that particularly targeted women with PCOS, 12 showed improvements in menstrual regularity and ovulatory function after bariatric surgery. Nine of these studies reported spontaneous conception within 12 to 24 months after surgery, while two reported partial hormonal changes but no pregnancy. Only one study found no significant reproductive changes in PCOS patients. A large proportion of the patients in these trials had polycystic ovarian syndrome (PCOS), which is a prominent cause of obesity-related infertility. The prevalence of PCOS varied amongst investigations, ranging from 13% in Gosman et al. (2010) to 100% in Eid et al. (2005), Jamal et al. (2012), and Ezzat et al. (2021). PCOS was significantly linked to anovulation, menstrual abnormalities, and hyperandrogenism, but studies consistently found significant improvements in ovulatory function and menstrual cycle control after bariatric surgery. Samarasinghe et al. (2024) found that 93% of patients with menstrual disorder had complete symptom relief within six months of surgery, whereas Jamal et al. (2012) found that PCOS patients had a 100% conception rate after RYGB. These data indicate that bariatric surgery might be one of the most successful treatments for PCOS-related infertility, owing to improvements in insulin sensitivity, androgen levels, and ovarian function. However, it is crucial to emphasize that PCOS is not the only condition that influences fertility in obese women. Some research, such as Christofolini et al. (2013) and Grzegorczyk-Martin et al. (2020), discovered that male factor infertility, tubal illness, and idiopathic reasons were common among post-bariatric surgery patients. This emphasizes the importance of complete infertility examinations and possibly assisted reproductive therapies for people who do not conceive naturally following surgery.

### 3.5. Fertility, Endocrine Outcomes, and Vitamin Deficiencies

Table 4 shows that bariatric surgery leads to considerable reproductive improvements, notably in patients with obesity-related infertility and polycystic ovary syndrome. A total of 21 out of 26 studies on fertility restoration after bariatric surgery found positive results in terms of spontaneous conception, hormonal normalization, or better reproductive symptoms. Furthermore, some studies found that menstrual dysfunction was completely resolved, such as Jamal et al. (2012), in which 82% of patients experienced restored menstrual cycles after RYGB, and Samarasinghe et al. (2024), in which 93% of patients with menstrual irregularities had full resolution within six months. However, not all trials showed benefits in fertility results. Studies by Legro et al. (2012), Neto et al. (2012), and Christofolini et al. (2013) discovered that bariatric surgery did not significantly improve fertility in respective patient groups. This diversity shows that fertility restoration may be influenced by additional factors such as baseline metabolic state, the degree of endocrine dysfunction, and post-surgical weight reduction effectiveness.

Bariatric surgery has a significant impact on hormone control, which affects fertility. The table demonstrates that various endocrine markers, including anti-Müllerian hormone (AMH), luteinizing hormone (LH), follicle-stimulating hormone (FSH), estradiol (E2), testosterone, and androstenedione, changed significantly after surgery. One of the most constant outcomes across trials was a drop in androgen levels, which is especially important for women with PCOS, a disorder characterized by hyperandrogenism. Eid et al. (2014), Nilsson-Condori et al. (2018), and Ezzat et al. (2021) found that post-surgery testosterone levels dropped significantly, resulting in improved ovulatory function and fewer symptoms like hirsutism. Similarly, Samarasinghe et al. (2024) found a substantial decrease in testosterone and androstenedione levels, indicating a favorable hormonal shift supporting increased fertility. However, certain endocrine indicators demonstrated inconsistent results among investigations. While some studies found that LH levels increased after surgery, others found that they remained steady or fell somewhat. For example, Eid et al. (2014) discovered that LH levels increased after RYGB, although Samarasinghe et al. (2024) showed no significant drop in LH levels after surgery. These data indicate that individual hormonal responses to bariatric surgery may vary depending on baseline reproductive function, the kind of surgery, and the degree of weight reduction accomplished. Another interesting observation is the continuous drop in AMH levels following surgery, as reported by Nilsson-Condori et al. (2018), Sahab Al Kabbi et al. (2018), Vincentelli et al. (2018), and Samarasinghe et al. (2024). AMH is a sign of ovarian reserve, and its decrease following bariatric surgery raises concerns about potential long-term reproductive consequences, particularly in women attempting conception later in life. The therapeutic ramifications of this drop are unknown, as some women continue to conceive naturally despite reduced AMH levels. It does, however, advise that patients undergoing bariatric surgery, particularly those who intend to postpone childbearing, be informed on fertility preservation alternatives.

A major issue following bariatric surgery is the danger of vitamin and micronutrient deficiencies, which can have a severe impact on pregnancy outcomes and fetal development. The table lists various studies that found deficits in iron, vitamin B12, folic acid, and vitamin D after surgery. One of the most often reported deficits was iron deficiency anemia, which can cause exhaustion, decreased oxygen transfer to reproductive organs, and pregnancy problems. Sheiner et al. (2004) recognized iron, folate, and B12 deficiencies as a substantial post-surgical problem, but Snoek et al. (2023) and Joly et al. (2024) discovered that vitamin shortages were more evident in patients following Roux-en-Y gastric bypass (RYGB) compared to sleeve gastrectomy. Similarly, Samarasinghe et al. (2024) discovered iron (8%), folic acid (19%), and vitamin D (14%) deficits in post-surgical patients, highlighting the importance of nutritional surveillance and supplementation in women of reproductive age. Yau et al. (2017) found that 65% of patients in the <2-year post-surgery group and 87% in the >2-year post-surgery group had vitamin D insufficiency. Vitamin D is essential for hormone balance and endometrial receptivity, and a lack of it can have a severe influence on fertility and pregnancy maintenance.

The table also gives information on miscarriage rates following bariatric surgery, which varied significantly between trials. Several studies found that surgery reduced miscarriage rates, particularly among women with obesity-related infertility. For example, Bilenka et al. (1995) discovered that miscarriage rates fell from 38.9% pre-surgery to 7.1% post-surgery, whilst Alatishe et al. (2013) observed a drop in miscarriage rates from 20.7% pre-surgery to 3.6% post-surgery. These data indicate that bariatric surgery may increase pregnancy viability by improving the mother’s metabolic health and lowering inflammation. However, several studies found greater rates of miscarriage following surgery, raising worries about potential consequences. Marceau et al. (2004) discovered that miscarriage rates increased from 21.6% pre-surgery to 26% post-surgery, but Goldman et al. (2016) observed that post-RYGB pregnancies had higher miscarriage rates than controls (OR = 9.81). Similarly, Cruz et al. (2019) discovered that miscarriage rates varied by time interval post-surgery, with the greatest rates occurring in the group that conceived between 12 and 24 months post-surgery (47.6%). These data indicate that, while bariatric surgery may increase fertility, it is critical to monitor patients for possible pregnancy concerns, especially in the early post-surgical period.

### 3.6. Pregnancy Complications Following Bariatric Surgery

Table 5 displays information on pregnancy-related issues in people who conceived after having bariatric surgery. Problems evaluated include intrauterine growth restriction (IUGR), gestational diabetes mellitus (GDM), preeclampsia, congenital abnormalities, and other pregnancy-related outcomes such as cesarean delivery rates, neonatal intensive care unit (NICU) admissions, and neonatal problems. The chart compares the occurrence of these issues before and after surgery, allowing us to determine if bariatric surgery decreases, increases, or has no influence on maternal and fetal health risks during pregnancy.

Bariatric surgery has a significant influence on pregnancy outcomes, including mother and fetal health. The data in this table show a complicated interplay between weight reduction, metabolic improvements, and probable dietary inadequacies, all of which can impact pregnancy problems. The most notable changes include lower rates of gestational diabetes mellitus (GDM) and preeclampsia, while concerns remain about increased risks of intrauterine growth restriction (IUGR), small-for-gestational-age (SGA) infants, and micronutrient deficiencies that may contribute to fetal development problems.

One of the most notable post-surgery advantages is a decrease in gestational diabetes. Studies consistently found a lower incidence of GDM, as predicted given the improvements in insulin sensitivity and weight loss after bariatric surgeries. Bilenka et al. (1995) and Jacamon et al. (2020) found significant decreases in GDM rates in post-surgical patients compared to obese controls, with the latter research reporting a drop from 44% in controls to 12% in the post-surgical group. Karadag et al. (2020) confirmed these findings, showing that GDM rates were considerably lower in women who underwent bariatric surgery than in the control group. However, certain studies, such as those conducted by Snoek et al. (2023) and Joly et al. (2024), found no significant differences in GDM risk, indicating that pre-pregnancy BMI, surgery type, and weight recovery may all affect outcomes. Despite these differences, the overall trend suggests that bariatric surgery provides significant protection against GDM, which is critical for minimizing perinatal problems and long-term metabolic abnormalities in children.

The effect of bariatric surgery on hypertensive diseases during pregnancy, notably preeclampsia, appears to be mixed. Several studies have found a decrease in preeclampsia rates following surgery, albeit the amount of this impact varies. Karadag et al. (2020) discovered that preeclampsia rates were reasonably steady among research groups, suggesting that, while bariatric surgery does not remove the risk of hypertensive problems, it does not necessarily aggravate them. Yau et al. (2017) found identical rates of preeclampsia in individuals who conceived within two years of surgery and those who conceived later, supporting the notion that other variables, such as pre-existing metabolic disorders and prenatal weight gain, may play a more important role. Sheiner et al. (2011) found that individuals who conceived within 12 months of surgery had a slightly higher preeclampsia rate than those who conceived later, indicating that the timing of conception may increase hypertension risks. This data supports concerns that early post-surgical pregnancies may be associated with persistent metabolic instability, thereby increasing the risk of hypertensive diseases.

Another important finding from these trials is an increased risk of intrauterine growth restriction (IUGR) after bariatric surgery. Fetal growth restriction is a prominent issue in pregnancies after malabsorptive surgeries because nutritional deficits can impair placental function and fetal development. Marceau et al. (2004) discovered a considerable rise in IUGR rates following surgery, going from 3.1% to 9.6%. This rise shows that, while bariatric surgery improves overall maternal metabolic health, it may result in poorer fetal development due to lower maternal food availability. However, other studies found no consistent rise in IUGR rates. Sheiner et al. (2011) found no significant differences in IUGR between pregnancies within 12 months after surgery and those happening later. Similarly, Facchiano et al. (2012) reported just a few incidences of IUGR in patients who underwent laparoscopic adjustable gastric banding (LAGB) and laparoscopic Roux-en-Y gastric bypass (LRYGB). These contradicting findings show that individual patient characteristics such as weight loss, malabsorption, and nutritional supplements adherence may have a major impact on IUGR risk.

Several studies have investigated the incidence of congenital abnormalities after surgery, however, the results remain ambiguous. Marceau et al. (2004) discovered an increase in congenital malformations from 2.6% pre-surgery to 4.2% post-surgery, raising questions about whether maternal micronutrient deficits influence aberrant fetal development. Given the critical function of folic acid, vitamin B12, and iron in embryonic neurodevelopment, any post-surgical shortages in these nutrients may increase the risk of congenital abnormalities. However, Sheiner et al. (2011) found no significant difference in congenital malformation rates between individuals who conceived within 12 months of surgery and those who conceived later, indicating that surgical weight reduction is not intrinsically teratogenic.

Aside from maternal problems, other research investigated neonatal outcomes, including the risk of small-for-gestational-age (SGA) newborns and NICU hospitalizations. Post-bariatric surgery pregnancies have been associated with an increased risk of SGA infants, likely due to reduced maternal nutrient availability and altered placental function. Gosman et al. (2010) discovered that post-surgical patients had much higher stillbirth rates than the general U.S. population, suggesting that high-risk pregnancies require close monitoring. Jacamon et al. (2020) and Snoek et al. (2023) discovered an elevated incidence of SGA newborns and NICU hospitalizations in post-surgical pregnancies, especially among women who received Roux-en-Y gastric bypass (RYGB). Similarly, Joly et al. (2024) found that SGA rates were greater among women who had sleeve gastrectomy vs. those who underwent gastric bypass, showing that different types of bariatric surgeries may carry differing degrees of fetal growth restriction risk.

The timing of conception after surgery appears to have an important effect on infant health. Cruz et al. (2019) discovered that newborn problems were most common among women who conceived within 12 months of surgery, with rates as high as 30%, as opposed to much lower rates in those who conceived later. This research implies that babies developing shortly after bariatric surgery may be more prone to difficulties due to persistent metabolic instability, fast weight reduction, and probable nutritional shortages. The increased risk of fetal growth restriction and newborn problems emphasizes the significance of postponing conception for at least 12 to 18 months after surgery to allow for maternal metabolic stability and appropriate nutritional replenishment.

Several studies included in this evaluation reported reproductive and perinatal outcomes based on the timing of conception after bariatric surgery. Pregnancies conceived within 12 months of surgery were more frequently associated with adverse outcomes, including higher rates of miscarriage, small-for-gestational-age (SGA) neonates, and maternal nutritional deficiencies, especially in cases of rapid weight loss and inadequate supplementation. Pregnancies between 12 and 18 months after surgery have been linked to better maternal metabolic profiles, fetal growth, and fewer problems. These findings back up current clinical guidelines that suggest delaying conception for at least 12 months following bariatric surgery. However, it is crucial to highlight that not all studies documented this timing clearly, and there is significant variation in how periods were classified, which limits direct comparisons.

A number of studies included in this evaluation described maternal and newborn problems after bariatric surgery. Micronutrient deficits were among the most common maternal concerns, especially in women who conceived during the first year after surgery or underwent malabsorptive surgeries like Roux-en-Y gastric bypass. The most common deficits found were iron, vitamin B12, folic acid, calcium, and fat-soluble vitamins, with some studies indicating the necessity for intravenous treatment during pregnancy. The deficiencies were linked to an increased risk of anemia, IUGR, spontaneous miscarriage, and low birth weight. Small-for-gestational-age (SGA) births and premature delivery were also common neonatal problems, particularly when conception occurred prior to nutritional stability. While long-term offspring follow-up was restricted, two studies found possible links with lower birth weight, shorter nursing duration, and marginally changed neurodevelopmental outcomes; nonetheless, the findings were varied and inconclusive.

## 4. Discussion

The included studies’ findings provide important insights into the effects of bariatric surgery on fertility, maternal outcomes, and neonatal health. The findings show that bariatric surgery dramatically improves fertility, especially in women with prior anovulatory dysfunction or polycystic ovarian syndrome (PCOS). Several studies in our study found a high rate of spontaneous ovulation and menstrual cycle normality after weight loss, resulting in improved conception rates without the use of assisted reproductive technology (ART). However, the evidence addressing ovarian reserve post-surgery is equivocal, with some studies revealing a decrease in anti-Müllerian hormone (AMH) levels, which could indicate a restricted reproductive window.

In terms of maternal outcomes, our findings show that bariatric surgery lowers the risk of obesity-related pregnancy problems, specifically gestational diabetes mellitus (GDM), hypertensive disorders, and preeclampsia. Women who had bariatric surgery had lower rates of gestational hypertension and preeclampsia, which supports the metabolic benefits of surgical weight loss. Despite these advantages, maternal nutritional deficiencies remained a major problem, particularly among Roux-en-Y gastric bypass (RYGB) patients, who frequently lacked iron, vitamin B12, folate, and vitamin D. The risk of maternal anemia was much higher in this group, underscoring the necessity of preconception nutritional optimization and ongoing monitoring during pregnancy.

The study of newborn outcomes found both beneficial and negative impacts of bariatric surgery on prenatal development. While macrosomia and large-for-gestational-age (LGA) newborns were considerably reduced, small-for-gestational-age (SGA) neonates and preterm births increased, particularly among RYGB patients. These data indicate that malabsorptive surgeries may have a deleterious impact on fetal growth, possibly due to decreased placental nutrition transfer and maternal micronutrient shortages. NICU admissions were more common in post-bariatric pregnancies, reflecting the increased susceptibility of the neonates born to these moms.

When comparing different types of bariatric surgery, our findings show that RYGB was associated with a greater reduction in metabolic complications but a higher risk of fetal growth restriction, whereas sleeve gastrectomy (SG) resulted in fewer nutritional deficiencies while still providing significant improvements in metabolic health. Studies that directly examined RYGB and SG found that the RYGB group had greater rates of SGA and neonatal problems, whereas SG appeared to give a better mix of metabolic advantages and fetal development results. Overall, our data underscore the complex trade-off between the benefits of bariatric surgery in lowering obesity-related pregnancy problems and the hazards of maternal malnutrition and fetal growth restriction. This emphasizes the importance of individualized preconception counseling, improved prenatal nutritional support, and long-term neonatal follow-up for women who conceive following bariatric surgery.

### 4.1. Bariatric Surgery and Fertility: The Restoration of Ovulatory Function and Enhanced ART Success

Obesity is a primary cause of female infertility, primarily because it disrupts the hypothalamic–pituitary–ovarian (HPO) axis, resulting in anovulation, irregular menstrual cycles, and metabolic dysfunction [56]. Our findings, which are consistent with the literature, show that bariatric surgery dramatically improves fertility, with a high rate of spontaneous ovulation, menstrual cycle stabilization, and better reproductive outcomes. Deitel et al. (1988) found that bariatric surgery improved monthly regularity and ovulation in women, indicating that weight loss directly affects reproductive health [22]. Samarasinghe et al. (2024) found that surgical weight loss effectively restored ovulatory periods for 93% of women experiencing preoperative menstrual disorder within six months [54]. Eid et al. (2005) found that women with polycystic ovarian syndrome (PCOS) who had bariatric surgery experienced a significant increase in spontaneous ovulation and pregnancy [26].

The underlying processes of reproductive improvement after bariatric surgery include major endocrine changes. Legro et al. (2012) found that higher levels of sex hormone-binding globulin (SHBG) and lower levels of free testosterone and estradiol promote a healthier reproductive hormonal environment. While these alterations increase ovulatory function, there have been concerns about ovarian reserve [33]. According to Sahab Al Kabbi et al. (2018), bariatric surgery dramatically reduced anti-Müllerian hormone (AMH) levels, a crucial marker of ovarian reserve. This suggests that women may have a shorter reproductive lifespan after surgery. Bariatric surgery has also been shown to benefit women who require assisted reproductive technology (ART) [42]. Grzegorczyk-Martin et al. (2020) observed that women who underwent bariatric surgery had superior embryo yields and fertilization rates compared to BMI-matched obese controls. This suggests that weight loss promotes ovarian responsiveness to controlled ovarian hyperstimulation (COH) [47]. This conclusion emphasizes the need for additional research into the long-term reproductive implications of bariatric surgery, specifically its impacts on oocyte quality, endometrial receptivity, and implantation rates.

### 4.2. Maternal Outcomes: Metabolic Benefits and Nutritional Risks

Bariatric surgery is well known for its significant metabolic benefits, notably in lowering obesity-related pregnancy problems such as gestational diabetes mellitus (GDM), hypertensive disorders, preeclampsia, and gestational hypertension [57]. The current study indicates that bariatric surgery greatly reduces the incidence of certain metabolic abnormalities, which is consistent with previous research showing the beneficial effects of surgical weight loss during pregnancy [58]. However, in addition to these benefits, the findings highlight the nutritional problems and deficits that occur after bariatric surgery, particularly in Roux-en-Y gastric bypass (RYGB) patients, due to the procedure’s malabsorptive nature [59].

One of the most convincing reasons for performing bariatric surgery on reproductive-aged women is the capacity to lower the risk of gestational diabetes mellitus and hypertensive disorders, two of the most prevalent and significant pregnancy problems linked to maternal obesity [20]. Obesity is a well-known risk factor for insulin resistance, hyperinsulinemia, and systemic inflammation, all of which contribute to the pathogenesis of gestational diabetes and hypertensive problems during pregnancy [60]. By causing weight loss and increasing metabolic function, bariatric surgery significantly reduces the prevalence of these disorders.

Our findings demonstrate that women who have had bariatric surgery have much lower rates of GDM than non-surgically obese controls. Johansson et al. (2015) found that post-bariatric surgery patients had a 72% reduced probability of acquiring GDM than non-surgical obese controls (1.9% vs. 6.8%), underscoring the metabolic benefits of weight loss [61]. Mustafa et al. (2023) observed that RYGB was associated with a 56% reduction in GDM risk and a 46% reduction in hypertensive disorders, supporting the importance of bariatric surgery in improving pregnancy-related metabolic health [62]. Kjaer et al. (2013) found that women who had previously undergone bariatric surgery had considerably decreased incidence of gestational hypertension and preeclampsia, highlighting the positive influence of surgical weight loss on cardiovascular health [63].

A similar pattern was identified for hypertensive diseases, such as preeclampsia and gestational hypertension. Marceau et al. (2004) reported that bariatric surgery dramatically reduced the rate of preeclampsia [25]. Menke et al. (2019) found that bariatric surgery significantly reduced hypertension problems compared to obese controls, demonstrating the cardiovascular benefits of large weight loss. The decrease in hypertensive diseases is most likely due to improved insulin sensitivity, endothelial function, and systemic inflammation, all of which contribute to the pathophysiology of pregnancy-induced hypertension. Despite these benefits, the best time to conceive after bariatric surgery is still contested, as earlier conception (within 12 months of surgery) may bring significant hazards [46]. According to Karadag et al. (2020), pregnancies within 12 months of bariatric surgery increase the risk of small-for-gestational-age (SGA) infants and lower birth weights. Waiting at least 18–24 months before conception may improve maternal metabolic stabilization and fetal development [49]. Yau et al. (2017) found that pregnancies occurring soon after surgery had increased risks of gestational hypertension and preeclampsia. This highlights the need for preconception counseling and the timing of pregnancy post-surgery [40].

Despite considerable metabolic improvements, nutritional shortages after bariatric surgery are still a serious problem, especially in malabsorptive operations like RYGB [27]. The changed morphology of the gastrointestinal system caused by RYGB results in decreased absorption of key micronutrients such as iron, vitamin B12, folate, vitamin D, and calcium, which can have major consequences for both maternal health and fetal development [64]. Our data show that maternal anemia and micronutrient deficiencies are common among post-bariatric surgery patients, stressing the importance of ongoing nutritional monitoring and supplementation. Snoek et al. (2021) discovered that women who conceived following bariatric surgery had considerably reduced levels of vitamin B12, iron, and folate, which raised the risk of maternal anemia [58]. Han et al. (2020) found that iron and folate shortages were more common in RYGB patients than in those undergoing sleeve gastrectomy (SG), raising concerns about the possibility of anemia and fetal growth restriction after malabsorptive surgeries [65].

Iron deficiency anemia (IDA) is especially concerning because it has been linked to premature birth, low birth weight, and increased perinatal morbidity. Thornton et al. (2021) found that post-bariatric pregnancy was linked to increased risk of maternal anemia, especially in women who did not obtain enough iron supplementation [51]. Joly et al. (2024) discovered that maternal anemia was more likely in women who had undergone RYGB compared to those with SG, emphasizing the necessity for personalized supplementing measures. In addition to iron and vitamin B12, folate deficiency is a major risk after bariatric surgery. Folate is required for fetal neural tube development, and low amounts raise the risk of neural tube abnormalities (NTDs) [53]. Samarasinghe et al. (2024) observed that women who underwent RYGB had reduced folate levels, despite routine prenatal treatment, indicating that greater doses of folic acid may be necessary in bariatric patients. Vitamin D deficiency is another major problem among post-bariatric patients [54]. Musella et al. (2011) discovered that women who underwent bariatric surgery had considerably reduced vitamin D levels during pregnancy, perhaps leading to poor fetal bone mineralization and neonatal rickets [30]. Yau et al. (2017) discovered greater rates of vitamin D deficiency in pregnancies following RYGB, highlighting the necessity for supplementation in this population. Given these findings, nutritional monitoring and supplements should be an important part of preconception care for post-bariatric surgery patients [40]. Thornton et al. (2021) suggest that post-bariatric women may require specialist bariatric pregnancy supplements regimens with increased iron, vitamin B12, folate, and vitamin D levels [51].

### 4.3. Neonatal Outcomes: The Challenge of Fetal Growth Restriction and Developmental Health

The influence of bariatric surgery on neonatal outcomes remains a major concern, particularly in terms of fetal growth restriction, birth weight anomalies, preterm birth, and long-term developmental health [66]. While maternal metabolic improvements after bariatric surgery help to reduce obesity-related pregnancy problems, there is growing evidence that these advantages come at the expense of fetal growth and newborn well-being. Our findings, along with previous research, point to a higher incidence of small-for-gestational-age (SGA) infants, preterm deliveries, and increased neonatal intensive care unit (NICU) admissions in pregnancies following bariatric surgery, particularly after malabsorptive procedures such as Roux-en-Y gastric bypass (RYGB). Understanding the underlying causes of adverse newborn outcomes is critical for improving prenatal care and lowering risks.

One of the most serious neonatal issues after maternal bariatric surgery is the increasing prevalence of SGA newborns, which are defined as neonates with a birth weight less than the 10th percentile for gestational age [67]. The current findings show that SGA risk is much higher in pregnancies following RYGB than in sleeve gastrectomy (SG) or obese control groups. This finding is consistent with earlier research, indicating that maternal micronutrient shortages, altered placental function, and decreased nutritional availability to the fetus may all contribute to growth restriction. Our research revealed that SGA rates were considerably greater in RYGB pregnancies than in SG pregnancies. Joly et al. (2024) found that 26.2% of neonates born to moms who underwent SG were classified as SGA, compared to 22.2% in the RYGB group [53]. Mandrup Kjaer et al. (2013) discovered that infants born to mothers who underwent bariatric surgery had a 2.3-fold higher probability of being SGA than those born to non-surgical obese mothers [68]. These findings suggest that SGA remains a substantial issue after bariatric surgery, especially in patients receiving malabsorptive operations.

The underlying causes of prenatal growth restriction following bariatric surgery are still being investigated. According to Johansson et al. (2015), placental insufficiency and decreased uteroplacental blood flow may cause fetal growth restriction [69]. Grzegorczyk-Martin et al. (2020) found that post-bariatric pregnancies can lead to altered placental function, potentially affecting fetal nutrition [47]. Furthermore, dietary deficiencies, particularly in iron, vitamin B12, and folate, play an important role in fetal development, and their deficiency in post-bariatric pregnancies likely increases the risk of growth restriction. Given the significant link between maternal malnutrition and fetal growth limitation, improved prenatal nutritional support and regular fetal growth monitoring are essential components of care for post-bariatric pregnant patients.

### 4.4. Preterm Birth and Increased NICU Admissions

Another neonatal consequence of concern following maternal bariatric surgery is an increased chance of preterm birth, which is defined as delivery before 37 weeks of pregnancy [70]. The current findings demonstrate that preterm birth rates are higher in post-bariatric pregnancies, especially those that occur within a short time after surgery. Our findings are consistent with previous research demonstrating a link between maternal bariatric surgery and increased incidence of spontaneous and indicated preterm birth. According to Karadag et al. (2020), pregnancies within 12 months of bariatric surgery are more likely to result in preterm delivery than those occurring later [49]. Mustafa et al. (2023) found that premature birth rates were greater in RYGB pregnancies compared to SG and non-surgical controls. This suggests that malabsorption and micronutrient deficiency may contribute to preterm labor [62].

Furthermore, we discovered a greater rate of NICU hospitalizations in neonates born to post-bariatric moms. Jacamon et al. (2020) discovered that newborns from post-bariatric pregnancies were more likely to need NICU hospitalization, raising concerns regarding fetal growth limitation, respiratory issues, and early neonatal adaptation challenges [48]. Snoek et al. (2021) found a higher prevalence of newborn hypoglycemia and transient respiratory distress syndrome in neonates born to moms who underwent bariatric surgery. This suggests that maternal metabolic changes may impact neonatal adaptation [58].

Beyond immediate newborn outcomes, emerging research suggests that maternal bariatric surgery may have long-term effects on offspring metabolic health and developmental programming [71]. The intrauterine environment has a significant impact on an infant’s metabolic trajectory, and obesity and post-bariatric surgery-induced dietary deficits can change fetal epigenetic regulation [72]. Our findings raise concerns about the long-term metabolic health of children born to moms who have had bariatric surgery. According to Grandfils et al. (2019), infants exposed to maternal bariatric surgery in utero may experience altered metabolic programming, leading to insulin resistance, obesity, and cardiometabolic disorders later in life [73]. Similarly, Ezzat et al. (2021) observed that offspring of bariatric surgery patients demonstrated variations in body composition and metabolic function compared to newborns of non-surgical mothers. This suggests that dietary shortages during fetal development may have long-term implications. Research has also raised concerns regarding the neurodevelopmental consequences of infants born to post-bariatric moms [50]. Gunakan et al. (2019) found abnormalities in neurocognitive function and motor development in children born to RYGB women, indicating the possible impact of maternal malnutrition on prenatal brain development. However, long-term studies are required to properly understand these children’s developmental trajectories and evaluate whether early postnatal interventions can reduce possible dangers [45].

### 4.5. Comparing Roux-en-Y Gastric Bypass (RYGB) and Sleeve Gastrectomy (SG): Which Procedure Is Optimal?

Roux-en-Y gastric bypass (RYGB) and sleeve gastrectomy (SG) are the two most common bariatric surgeries performed on women of reproductive age [74]. Both methods result in significant weight loss and metabolic benefits, but they have different mechanisms of action, dietary repercussions, and pregnancy-related results. Understanding the relative benefits and dangers of each operation is critical for making informed surgical decisions, particularly for women planning future pregnancies [75]. While RYGB has generally been the preferred option due to its superior weight loss and metabolic benefits, new evidence suggests that SG may provide a better combination of maternal metabolic gains and fetal safety due to its less severe malabsorptive effects. Our findings, which are confirmed by previous research, indicate that RYGB may be more successful in reducing obesity-related pregnancy problems than SG, but it also increases the risk of fetal growth restriction and maternal malnutrition.

Both RYGB and SG are quite effective at lowering body weight and improving metabolic parameters such as glucose regulation, insulin sensitivity, and hypertension control [76]. The metabolic benefits of RYGB are more pronounced, notably in terms of type 2 diabetes remission and insulin resistance, which have significant implications for lowering gestational diabetes mellitus (GDM) and hypertensive problems during pregnancy [77]. Our findings confirm that RYGB reduces the incidence of GDM more than SG, which is consistent with previous research. According to Mustafa et al. (2023), RYGB reduced GDM risk by 56%, but SG did not demonstrate a significant reduction [62]. Menke et al. (2019) reported that RYGB patients had considerably lower incidences of gestational hypertension and preeclampsia compared to those undergoing SG, further supporting the cardiovascular benefits of RYGB [46]. Grandfils et al. (2019) found that RYGB significantly improved insulin sensitivity and glucose metabolism compared to SG, leading to a decreased incidence of pregnancy-induced metabolic problems [73].

Despite its improved metabolic benefits, RYGB carries higher nutritional risks, which may have a deleterious influence on fetal and maternal health. Samarasinghe et al. (2024) discovered that iron, vitamin B12, and folate shortages were more common in RYGB patients compared to SG patients, resulting in greater rates of maternal anemia and pregnancy problems. In contrast, SG is a strictly restrictive operation, which means that it does not cause malabsorption of critical micronutrients to the same extent as RYGB. This conserved nutrient absorption may guard against severe maternal nutritional shortages during pregnancy, while also providing significant weight reduction and metabolic benefits [54]. According to Joly et al. (2024), SG may be a safer alternative for pregnant women’s micronutrient status, as it resulted in fewer incidences of severe iron deficiency anemia than RYGB [53].

The main disadvantage of RYGB compared to SG is its high malabsorptive component, which raises the risk of nutritional deficiencies. Given how important maternal micronutrient levels are for fetal growth and development, these deficits can have substantial ramifications for both maternal and newborn health. Our findings, which are similar to earlier research, demonstrate that maternal iron, vitamin B12, folate, and vitamin D deficits are more common in RYGB patients than in SG patients. Han et al. (2020) found that RYGB patients had lower serum levels of iron, vitamin B12, and folate compared to SG patients, resulting in greater incidences of maternal anemia and fetal growth limitation [65]. Thornton et al. (2021) found that women with a history of RYGB needed more micronutrient supplementation than those with SG, as normal prenatal vitamins were generally insufficient [51].

Snoek et al. (2021) found that RYGB patients had considerably lower vitamin D levels during pregnancy than SG patients, perhaps leading to fetal bone mineralization difficulties, neonatal rickets, and long-term skeletal abnormalities. These data show that, while both operations necessitate thorough nutritional monitoring, RYGB patients are at a significantly higher risk of acquiring clinically relevant deficits. Given the nutritional concerns associated with RYGB, SG may be a better option for reproductive-aged women, especially those who are unable to carefully adhere to lifelong supplement regimens. However, SG is not without nutritional problems, as fast weight loss and reduced food consumption following surgery can still result in vitamin deficiency, necessitating frequent nutritional supervision and supplementation in both groups [58].

### 4.6. Fetal Growth Outcomes: The Risk of Small-for-Gestational-Age (SGA) Infant

One of the most serious neonatal issues linked with RYGB is an increased risk of small-for-gestational-age (SGA) newborns and fetal growth restriction, which are most likely caused by maternal malnutrition, placental insufficiency, and decreased fetal nutritional transfer [21].

Our findings, which are consistent with previous research, show that SGA risk is much higher in RYGB pregnancies than in SG pregnancies. Joly et al. (2024) found that 26.2% of neonates born to SG moms were classified as SGA, compared to 22.2% in the RYGB group [53]. According to Mandrup Kjaer et al. (2013), babies born after bariatric surgery have a 2.3-fold higher risk of having SGA than those born to non-surgical obese moms [68]. The fundamental mechanisms of fetal development limitation after RYGB are considered to involve:

- Reduced placental nutrition transfer causes intrauterine growth restriction (IUGR).
- Maternal anemia and iron deficiency might impede fetal oxygenation and growth.
- Vitamin B12 and folate deficits raise the risk of placental insufficiency and fetal malnutrition.

In contrast, SG seems to be related to a decreased incidence of SGA in babies. Grandfils et al. (2019) showed that SG patients had a lower rate of prenatal growth restriction than RYGB patients, indicating that intact nutrient absorption may play a protective role in fetal development [73].

These findings indicate that, whereas RYGB provides higher metabolic benefits, SG may strike a better balance between maternal health and baby safety by lowering the risk of severe micronutrient shortages and fetal development issues.

Despite the benefits of bariatric surgery for conception and pregnancy restoration, numerous studies have found significant maternal and newborn problems. Nutritional deficiencies, particularly iron, vitamin B12, folate, calcium, and fat-soluble vitamins, were prominent in malabsorptive surgeries and cases of pregnancy within the first year after surgery. These impairments were linked to an increased risk of miscarriage, hypertension, low birth weight, and newborns that were short for their gestational age (SGA). Furthermore, a small number of studies have expressed concerns about long-term repercussions in kids, such as decreased birth weight, shorter breastfeeding length, and potential effects on neurodevelopment, however, evidence is sparse. These findings emphasize the significance of thorough nutritional monitoring and postponing conception until stability has been reached.

## 5. Future Research Directions and Clinical Recommendations

Bariatric surgery has considerably improved maternal metabolic health by lowering obesity-related problems such as gestational diabetes, preeclampsia, and hypertensive diseases. However, worries about maternal nutritional deficits, fetal growth limitation, and long-term offspring health continue. Future research should concentrate on improving clinical recommendations to ensure maternal and newborn safety while optimizing the benefits of bariatric surgery.

One of the most significant research requirements is to better understand the metabolic and developmental effects of children born to post-bariatric moms. While bariatric surgery lowers the dangers associated with maternal obesity, new research reveals that in utero exposure to rapid maternal weight reduction and altered food absorption may predispose kids to metabolic diseases such as insulin resistance and obesity later in life. Longitudinal cohort studies are required to study these children’s health throughout adolescence and adulthood in order to discover whether early maternal dietary interventions can reduce these risks. Another critical area of study is the impact of placental function in fetal growth restriction following bariatric surgery. It is unknown whether lower fetal growth is predominantly caused by maternal micronutrient deficits, placental insufficiency, or a combination of the two. Studies on placental shape, vascularization, and nutrient transport dynamics in post-bariatric pregnancies are critical for designing tailored therapies that improve fetal growth outcomes while preserving maternal metabolic health.

Nutritional supplementation is a critical component of post-bariatric pregnancy care, although there is no universal agreement on the best supplementation plan. Current information suggests that normal prenatal vitamins may not be adequate to address the nutritional demands of post-bariatric women, particularly those who have undergone Roux-en-Y gastric bypass surgery. Future research should look into the optimal quantities and formulations of iron, vitamin B12, folate, and vitamin D to prevent maternal deficits and increase fetal health. Research should also look into whether individualized supplementation based on trimester-specific nutritional assessments can better address pregnant women’s changing needs following bariatric surgery. Another important subject that requires additional exploration is the best time of pregnancy after bariatric surgery. Current guidelines advocate delaying conception for at least 12 to 18 months after surgery, but new research suggests that extending this interval to 24 months may further minimize the risk of fetal growth restriction and preterm birth. More study is needed to discover whether postponing pregnancy beyond 18 months has additional maternal and newborn benefits, or whether focused nutritional interventions can enhance outcomes for women who conceive earlier.

Comparative studies of Roux-en-Y gastric bypass and sleeve gastrectomy in pregnant women are also required to determine the optimum surgical strategy for reproductive-aged women. While Roux-en-Y gastric bypass provides improved metabolic benefits and greater weight loss, it is also linked to an increased risk of maternal nutritional shortages and fetal development restriction. Sleeve gastrectomy, on the other hand, appears to strike a balance between metabolic benefits and a lower risk of malnutrition, making it a potentially safer option for pregnancy. Large-scale prospective studies comparing the pregnancy and neonatal outcomes of these two operations will provide useful information for personalizing surgical recommendations to women of reproductive age. Preconception counseling should be an important part of care for women who have had bariatric surgery. A thorough preconception evaluation should consider metabolic stability, nutritional condition, and potential surgical problems that could jeopardize pregnancy. Preconception nutritional screening should include a check of iron, vitamin B12, folate, and vitamin D levels to ensure that any deficiencies are addressed before conception. Women should also be counseled on the potential dangers and benefits of pregnancy after bariatric surgery, such as the significance of postponing conception until weight loss has stabilized and nutritional status has been maximized.

Women who have had bariatric surgery should undergo specific prenatal care, such as routine fetal growth monitoring and nutritional assessments. Serial ultrasounds are recommended to monitor fetal growth and detect any early signs of intrauterine growth limitation. Nutritional assessments should be performed every trimester to ensure that maternal iron, vitamin B12, folate, and vitamin D levels are within the recommended range. Given the high prevalence of maternal anemia in post-bariatric pregnancies, proactive iron supplementation is recommended, especially for Roux-en-Y gastric bypass patients, who are at the highest risk of iron deficiency anemia.

Traditional oral glucose tolerance tests may not be well tolerated by post-bariatric individuals, necessitating a modification of gestational diabetes screening. Alternative screening approaches, such as continuous glucose monitoring or fasting glucose and hemoglobin A1c tests, should be explored for diagnosing gestational diabetes in this population.

After delivery, postpartum nutritional care is essential for maternal recovery and optimal nursing nutrition. Women should continue to obtain personalized supplements to rebuild nutritional stores that were reduced during pregnancy. Long-term pediatric follow-up for kids should be addressed, especially in cases of maternal nutritional deficits or fetal growth limitation during pregnancy. Children born to post-bariatric moms should be evaluated for metabolic health, growth anomalies, and neurodevelopmental outcomes in order to detect any problems early and give appropriate treatment. Given the increasing number of bariatric procedures performed in reproductive-aged women, a multidisciplinary approach to care is required. Obstetricians, bariatric specialists, dietitians, and pediatricians should work together to create tailored care plans that improve maternal health while also assuring the best potential outcomes for newborns. Standardized methods for prenatal nutritional screening, gestational diabetes monitoring, and fetal growth assessment in post-bariatric pregnancies should be created to ensure consistent and evidence-based care.

Future studies should also focus on developing long-term maternal health standards after bariatric surgery and pregnancy. Many post-bariatric women face chronic dietary problems after pregnancy, making long-term follow-up essential. Understanding how pregnancy affects long-term weight maintenance, metabolic health, and surgical outcomes will help optimize care for these women during their reproductive years and beyond. Finally, while bariatric surgery has the potential to enhance maternal health and reduce pregnancy difficulties caused by obesity, careful monitoring is essential to address nutritional inadequacies and fetal growth concerns. Achieving a balance between maternal metabolic health and fetal well-being will require ongoing research, improved clinical practices, and a collaborative multidisciplinary approach.

Bariatric surgery’s favorable benefits on female fertility are supported by both clinical and molecular research. Substantial weight loss following surgery improves insulin sensitivity, which reduces ovarian androgen production and aids in the restoration of the hypothalamic–pituitary–ovarian (HPO) axis. Bariatric surgery reduces systemic inflammatory indicators such as CRP, IL-6, and TNF-α, which can affect ovulation and implantation. Hormones like leptin and ghrelin, which are considerably altered after surgery, have also been demonstrated to influence GnRH pulsatility and follicular development. Changes in anti-Müllerian hormone (AMH) levels demonstrate enhanced ovarian response following weight loss. Together, these endocrine, metabolic, and inflammatory alterations contribute to the reported clinical improvements in reproductive indices throughout the trials.

While existing research suggests that bariatric surgery improves fertility and pregnancy outcomes in obese women, numerous crucial knowledge gaps remain. These include the best time to conceive after surgery, procedure-specific reproductive outcomes, and the long-term implications on offspring health and development. Future research should concentrate on prospective, multicenter trials with uniform fertility endpoints and long-term follow-up to address these unsolved problems and improve evidence-based reproductive care guidance.

Our findings are partially similar to prior systematic reviews, but they give a more detailed clinical classification. For example, Makhsosi et al. (2024) found a pooled post-surgical conception rate of 67% among obese infertile women, confirming a clear link between bariatric surgery and fertility restoration [78]. Similarly, Al Qurashi et al. (2022) discovered significant increases in sex hormone levels and sexual performance in both men and women after bariatric surgery, particularly with regard to weight loss and insulin sensitivity [79]. However, these trials were primarily concerned with overall fertility restoration and did not include in-depth examinations of pregnancy problems, conception timing, or neonatal outcome.

In contrast, our study focuses on key clinical details that were not adequately addressed in the previous papers. We examined papers stratified by the time interval between surgery and conception and found that complications were more common in pregnancies occurring within 12 months of surgery. Furthermore, we included data on maternal nutritional inadequacies, such as iron, B12, and folate deficiency, which have been linked to small-for-gestational-age newborns and an increased risk of miscarriage. Finally, our synthesis incorporated the limited but expanding body of information on long-term offspring outcomes, which had not been covered by prior meta-analyses. Taken together, these factors enable our review to provide more detailed information to clinicians counseling women of reproductive age having bariatric surgeries.

## 6. Limitations

Despite the thorough methods used in this systematic review, many limitations should be noted to guarantee a fair interpretation of the findings. One of the main drawbacks is the variability of the included studies in terms of research design, surgical techniques, fertility results, and follow-up time. The study included retrospective and prospective cohort studies, as well as randomized controlled trials (RCTs), all of which differed in methodological quality and bias risk. Retrospective studies, which made up the bulk of the available literature, are naturally hampered by recollection bias, missing data, and discrepancies in medical record-keeping, which may impact the accuracy of reported fertility and pregnancy results.

Another issue is the heterogeneity in bariatric surgery procedures among the studies. Different surgical methods, such as Roux-en-Y gastric bypass (RYGB), sleeve gastrectomy (SG), adjustable gastric banding (AGB), and biliopancreatic diversion (BPD), have diverse metabolic and hormonal consequences that may affect reproductive results differently. However, due to a lack of direct comparison trials comparing reproductive outcomes across different procedures, it was not possible to stratify data by surgery type, potentially limiting the generalizability of the results. Inconsistencies in fertility outcome reporting between studies confound direct comparisons. Some studies measured menstrual cycle regularity, ovulatory function, or reproductive hormone levels, whilst others looked at conception rates, pregnancy outcomes, and newborn health markers. The lack of defined fertility outcome measures makes it difficult to conduct meta-analyses and reach consistent conclusions on the influence of bariatric surgery on reproductive function.

Furthermore, confounding variables such as age, pre-existing metabolic disorders (e.g., insulin resistance, polycystic ovarian syndrome [PCOS]), and the use of assisted reproductive technology (ART) were not consistently controlled for across research. Some studies controlled for these characteristics in their analysis, whereas others did not, which might have an impact on claimed fertility gains. The absence of uniform preoperative BMI and weight loss reporting further restricts the ability to link reproductive results to the degree of weight reduction accomplished after surgery.

Some studies had relatively short follow-up periods, with minimal information on long-term reproductive results beyond two years after surgery. Because bariatric surgery causes progressive metabolic and hormonal changes, a longer follow-up period is required to see whether reproductive benefits last over time. Furthermore, most studies did not include extensive assessments of long-term child health and developmental outcomes, making it impossible to assess the potential transgenerational impacts of a mother’s bariatric surgery. Another significant drawback is the possibility of publication bias, since studies that indicate favorable reproductive outcomes after bariatric surgery are more likely to be published than those with negative or ambiguous results. Due to the variety of research designs, no formal publication bias assessment (e.g., funnel plot, Egger’s test) was undertaken; nonetheless, this bias should be taken into account when interpreting the results.

Finally, while attempts were made to reduce bias in the quality evaluation, the use of the Newcastle–Ottawa Scale (NOS) for observational studies and the Cochrane Risk of Bias Tool for RCTs introduces some subjectivity. Although two separate reviewers used Cohen’s kappa analysis to establish inter-rater reliability, the rating of research quality still involves some subjective judgment. Despite these limitations, this systematic review sheds light on the relationship between bariatric surgery and female fertility outcomes, emphasizing the importance of additional well-designed, prospective studies with standardized outcome measures, longer follow-up periods, and comparative analyses across different surgical techniques to strengthen the evidence base.

## 7. Conclusions

Bariatric surgery has emerged as an important option for managing extreme obesity, particularly in reproductive-aged women, with considerable benefits in lowering obesity-related pregnancy problems such as gestational diabetes mellitus (GDM), preeclampsia, and hypertensive diseases. However, this review emphasizes the difficult balance between maternal metabolic benefits and potential dangers to fetal and newborn health. While Roux-en-Y gastric bypass (RYGB) and sleeve gastrectomy (SG) are both effective weight loss surgeries, they have different nutritional effects and affect pregnancy outcomes.

Our findings, which are validated by previous studies, show that while RYGB provides superior metabolic benefits, it also increases the risk of maternal micronutrient shortages, small-for-gestational-age (SGA) newborns, and fetal growth restriction. On the other hand, SG, while slightly less effective in metabolic illness remission, is associated with a lower risk of severe maternal malnutrition and neonatal growth abnormalities, making it a potentially safer surgical alternative for reproductive-aged women.

The increasing prevalence of SGA children, preterm birth, and neonatal intensive care unit (NICU) hospitalizations in post-bariatric pregnancies, particularly following malabsorptive treatments, raises serious concerns. Placental insufficiency, maternal anemia, and altered fetal nutrient transfer mechanisms may all contribute to these negative outcomes, demanding increased prenatal monitoring and tailored nutritional support. Given the hazards, comprehensive preconception counseling, serial fetal growth monitoring, and tailored nutritional supplementation should be included in normal post-bariatric pregnancy treatment.

Furthermore, growing research suggests that kids of bariatric surgery patients may face long-term metabolic and neurodevelopmental problems, most likely due to altered maternal–fetal nutrition exchange and epigenetic changes during gestation. These findings highlight the importance of long-term follow-up research on the metabolic and developmental health of children born to post-bariatric moms.

Clinically, managing pregnancy after bariatric surgery necessitates a collaborative effort by obstetricians, bariatric specialists, endocrinologists, and neonatologists. Standard prenatal screening measures, such as gestational diabetes tests, should be adapted to account for altered gut anatomy, while routine iron, vitamin B12, folate, and vitamin D levels must be monitored to avoid maternal and fetal issues. Given the increasing number of bariatric procedures performed on women of childbearing age, future research should concentrate on identifying appropriate surgical techniques, refining nutritional supplementation guidelines, and determining the long-term health implications on children. Large-scale prospective studies comparing RYGB with SG in pregnant women are required to make definitive recommendations about the safest and most beneficial surgery for women planning future pregnancies.

To summarize, while bariatric surgery remains an effective technique for combating obesity and its related reproductive issues, it is not without dangers. To optimize maternal and fetal health, a careful balance must be struck between the benefits of weight loss and the prevention of nutritional deficits. Continuous research, improved clinical practices, and a patient-centered interdisciplinary approach can lead to the greatest potential outcomes for both mother and child. This review validates and expands on previous findings by including more recent research, broadening the focus beyond PCOS groups, and summarizing the literature in a way that prioritizes clinical relevance. Rather than proposing a novel theoretical model, we aimed to provide a thorough and accessible summary that may be used to guide preconception counseling, reproductive planning, and risk assessment in women undergoing bariatric surgery.

Although formal publishing bias evaluation using funnel plots or Egger’s regression was not possible due to the lack of meta-analytic pooling, we recognize the possibility of publication bias. The majority of the examined studies showed good reproductive results following bariatric surgery, with only a few reporting null or detrimental effects. This pattern opens up the potential of selective publication or result reporting. Furthermore, the prevalence of single-center and retrospective designs might lead to reporting bias. Readers should evaluate the findings with these limitations in mind.

## Figures and Tables

**Table 1:** Quality Assessment of Included Studies in the Systematic Review on Bariatric Surgery and Female Fertility.

Author	Study Design	Sample Size	Selection Bias	Outcome Assessment	Follow-Up Duration	Risk of Bias	Overall Quality
Deitel et al., 1988 [22]	Retrospective	138	Moderate (strict inclusion criteria)	Well-defined, clear pregnancy outcomes	2–5 years	Moderate (retrospective design)	Moderate
Bilenka et al., 1995 [23]	Retrospective	9	High (small sample, limited data)	Well-defined fertility outcomes	Not reported	High (small cohort, potential recall bias)	Low
Sheiner et al., 2004 [24]	Retrospective	298	Low (large sample size)	Pregnancy outcomes well reported	Not reported	Moderate (retrospective nature)	Moderate
Marceau et al., 2004 [25]	Retrospective	132	Moderate (high response rate)	Well-defined fertility outcomes	2–18 years	Moderate (potential recall bias)	Moderate
Eid et al., 2005 [26]	Retrospective	24	Moderate (PCOS-focused)	Clear reproductive outcomes	Not reported	Moderate (small cohort)	Moderate
Rochester et al., 2009 [27]	Prospective	9	High (small sample, selection bias)	Clear eligibility criteria, but limited follow-up	6–12 months	High (small sample)	Low
Gosman et al., 2010 [28]	Retrospective	1538	Low (large sample)	Clear pregnancy outcomes	Not reported	Moderate	High
Sheiner et al., 2011 [29]	Retrospective	489	Low (large sample)	Well-defined pregnancy outcomes	Not reported	Moderate	High
Musella et al., 2011 [30]	Retrospective	23	Moderate (strict inclusion criteria)	Well-reported fertility outcomes	1–11 months	Moderate (small sample)	Moderate
Facchiano et al., 2012 [31]	Retrospective	36	Moderate	Clear reproductive outcomes	Not reported	Moderate	Moderate
Jamal et al., 2012 [32]	Retrospective	20	Moderate (PCOS focus)	Clear fertility and pregnancy outcomes	Not reported	Moderate	Moderate
Legro et al., 2012 [33]	Prospective	29	Moderate	Well-reported pregnancy rates	Up to 24 months	Moderate	Moderate
Neto et al., 2012 [34]	Retrospective	140	Low (good follow-up)	Well-defined infertility remission outcomes	5+ years	Moderate	High
Alatishe et al., 2013 [35]	Retrospective	232	Low	Well-reported pregnancy outcomes	Median 11 months	Moderate	High
Christofolini et al., 2014 [36]	Retrospective	94	Moderate (bariatric surgery group small)	No significant differences between groups	Not reported	Moderate	Moderate
Eid et al., 2014 [37]	Prospective	14	Moderate (small PCOS cohort)	Clear hormonal and menstrual outcomes	12 months	Moderate	Moderate
Goldman et al., 2016 [38]	Retrospective	219	Low	Well-defined pregnancy outcomes	<18 months	Moderate	High
Milone et al., 2017 [39]	Retrospective	40	Moderate	Well-reported pregnancy rates	≤24 months	Moderate	Moderate
Yau et al., 2017 [40]	Retrospective	54	Moderate	Well-defined pregnancy outcomes	Not reported	Moderate	Moderate
Nilsson-Condori et al., 2018 [41]	Prospective	48	Moderate	Clear AMH, FSH, and hormone level changes	12 months	Moderate	Moderate
Sahab Al Kabbi et al., 2018 [42]	Prospective	60	Moderate	Well-reported menstrual improvement	65 ± 5 days	Moderate	Moderate
Vincentelli et al., 2018 [43]	Retrospective	39	Moderate	AMH and hormone assessment well documented	12 months	Moderate	Moderate
Cruz et al., 2019 [44]	Retrospective	42	Moderate	Well-defined neonatal outcomes	Not reported	Moderate	Moderate
Gunakan et al., 2019 [45]	Retrospective	23	Moderate	Well-defined pregnancy outcomes	Not reported	Moderate	Moderate
Menke et al., 2019 [46]	Prospective	650	Low	High pregnancy rates post-surgery	<7 years	Low	High
Grzegorczyk-Martin et al., 2020 [47]	Retrospective	83	Moderate	IVF-related pregnancy rates	Mean 2.98 years	Moderate	Moderate
Jacamon et al., 2020 [48]	Retrospective	52	Moderate	Well-defined pregnancy outcomes	Median 3 years	Moderate	Moderate
Karadag et al., 2020 [49]	Retrospective	144	Low	Well-defined pregnancy and neonatal outcomes	25.8 months	Moderate	High
Ezzat et al., 2021 [50]	Prospective	36	Moderate	Clear PCOS infertility assessment	1 year	Moderate	Moderate
Thornton et al., 2021 [51]	Retrospective	460	Low	Well-defined pregnancy rates	18 months	Moderate	High
Snoek et al., 2024 [52]	Prospective	97	Moderate	Neonatal and maternal outcomes well documented	Median 18.3 months	Moderate	High
Joly et al., 2024 [53]	Retrospective	244	Low	Clear pregnancy outcomes	33.7 months (sleeve), 40.9 months (bypass)	Moderate	High
Samarasinghe et al., 2024 [54]	RCT	80	Low	Strong PCOS and fertility outcomes	12–18 months	Low	High

Table 1 evaluates the methodological quality of the studies included in the systematic review. Studies were assessed based on their research design, sample size, potential selection bias, outcome measurement, follow-up duration, risk of bias, and overall quality. A quality evaluation was performed to guarantee the reliability and validity of the findings given in the included studies. Retrospective studies were typically assigned a greater risk of bias due to probable recollection mistakes, but prospective and randomized controlled trials (RCTs) were regarded as having a reduced risk of bias due to improved control over confounding variables. Studies with bigger sample numbers and well-defined outcome evaluations were regarded as having higher overall quality, whereas those with small sample sizes, missing follow-up data, or unclear procedures were thought to be at moderate to high risk of bias.

**Table 2:** Methodological Characteristics.

Author	Type of Study	Sample Size	Inclusion Criteria	Exclusion Criteria	Type of Bariatric Surgery	Time Interval Between Bariatric Surgery and Pregnancy	Pregnancy Outcome
**Deitel et al., 1988 [22]**	Retrospective	138	- **Morbidly obese women** (≥45 kg over ideal weight, ≥2 × ideal weight) - **Underwent bariatric surgery** (jejunoileal bypass, horizontal/vertical banded gastroplasty) **- Lost ≥50% of excess weight** post-surgery - **Euthyroid preoperatively** (normal thyroid function) - **Complete gynecologic-obstetric data** pre- and post-surgery (2–5 years)	- **Weight loss < 50%** post-surgery - **Untreated thyroid disorders** - **Missing postoperative follow-up data**	Jejunoileal bypass (6), horizontal gastroplasty (23), vertical banded gastroplasty (109)	**- ≥18 months** after **jejunoileal bypass** **- ≥12 months** after **gastroplasty (horizontal or vertical banded)**	- **Total Pregnancies After Surgery: 9-**all **resulted in live births** - Infertility resolved in 8/9 patients
**Bilenka et al., 1995 [23]**	Retrospective	9	- Women who underwent **VBG** for morbid obesity - available **pre- and post-surgery reproductive history** - Age at the time of surgery: **24–36 years** (mean: **31.9 years**) - Mean weight loss post-surgery: **36 kg** (range: 19–55 kg) - At least **1 pregnancy** before or after surgery	Women - Who did not undergo VBG - Without **available reproductive history** - Who did not attempt pregnancy **before or after VBG** - **Other major medical conditions** interfering with pregnancy	**Vertical banded gastroplasty (VBG)**	N/A	**- Improved fertility. After VBG: 14 pregnancies** in **9 women.** **All conceived spontaneously** **- Miscarriage rates decreased significantly (7.1% after VBG vs. 38.9% before)** **- Pregnancy complications were lower** post-surgery. **- Infant outcomes were generally favorable**
**Sheiner et al., 2004 [24]**	Retrospective	298	Pregnancies with history of bariatric surgery	N/A	Restrictive and malabsorptive (open/laparoscopic)	N/A	No significant adverse outcomes; Higher CS rates
**Marceau et al., 2004 [25]**	Retrospective	132	Women who had successfully undergone biliopancreatic diversion (BPD)	- Who did not successfully undergo **BPD** surgery - Women who did not respond to the questionnaire (although response rate was high at 85.5%). - Women who had **BPD less than two years prior to the study** may have been excluded from certain analyses	Biliopancreatic diversion (BPD)	Varied; pregnancies occurred 2–18 years after surgery, with some within 2 years	Increased fertility post-surgery (47% of previously infertile women conceived)
**Eid et al., 2005 [26]**	Retrospective	24	Women with PCOS undergoing bariatric surgery	Incomplete follow-up data or medication use	Laparoscopic Roux-en-Y gastric bypass	N/A	All five infertile women conceived post-surgery without medication
**Rochester et al., 2009 [27]**	Prospective	9	Age 18–48, regular menstrual cycles (21–40 days), BMI ≥ 35 kg/m^2^, uterus and at least one ovary	Renal, hepatic, or systemic disease, exogenous hormone use in last 3 months, diabetes, alcoholism, PCOS	Gastric bypass (10), Lap-band (6)	N/A	N/A
**Gosman et al., 2010 [28]**	Retrospective	1538	Women undergoing bariatric surgery, age ≥ 18, no prior weight loss surgery	Women reporting tubal sterilization, or a husband/live-in partner who had a vasectomy, missing pregnancy history data, prior bariatric surgery	Gastric bypass (74.1%), adjustable gastric banding (23.2%), other (2.7%)	N/A	72.5% had at least one live birth, 25.2% had at least one miscarriage, 2.0% had at least one stillbirth
**Sheiner et al., 2011 [29]**	Retrospective	489	Women who conceived after bariatric surgery	Multiple gestations	Laparoscopic gastric banding (LAGB), silastic ring vertical gastroplasty (SRVG), vertical-banded gastroplasty (VBG), Roux-en-Y gastric bypass (RGB)	<12 months (mean 7.0 ± 3.5 months), >12 months (mean 56.7 ± 49.1 months)	104 conceived < 12 months post-op, 385 conceived > 12 months post-op; Comparable perinatal outcomes between groups, 1.9% congenital malformations in early group vs. 1.3% in late group
**Musella et al., 2011 [30]**	Retrospective	23	Obese women with infertility, undergoing intragastric balloon treatment	Infertile male partner (4 women excluded)	Intragastric balloon (BIB^®^ Bioenterics Intragastric Balloon)	1–11 months after weight loss	15/18 (83.3%) women who lost weight became pregnant; all pregnancies ended with live births and no complications
**Facchiano et al., 2012 [31]**	Retrospective	36 (19 with LAGB, 17 with LRYGB); 42 pregnancies	Women who underwent LAGB or LRYGB and later became pregnant	None	Laparoscopic adjustable gastric banding (LAGB) (19), laparoscopic Roux-en-Y gastric gypass (LRYGB) (17)	LAGB: 30 months LRYGB: 25 months	42 pregnancies. No significant difference between LAGB and LRYGB in obstetric or neonatal outcomes
**Jamal et al., 2012 [32]**	Retrospective	20	Morbidly obese women undergoing Roux-en-Y gastric bypass (RYGB), PCOS diagnosis	Postmenopausal women (6), lost to follow-up (5)	Roux-en-Y gastric bypass (RYGB)	N/A	100% conception rate in infertile PCOS patients post-surgery, no reported complications
**Legro et al., 2012 [33]**	Prospective	29	Women aged 18–40, BMI > 40 or BMI 35–39.9 with comorbidities, premenopausal, intact ovaries and uterus, no hormonal contraceptive use	Smoking, alcohol/substance abuse, pregnancy, unwillingness to use barrier contraception, metabolic disorders (hypothyroidism, Cushing’s syndrome, genetic predisposition)	Roux-en-Y gastric bypass (RYGB)	Up to 24 months	5 pregnancies post-surgery
**Musella et al., 2012 [55]**	Retrospective	110	Obese women with infertility, undergoing bariatric surgery	Infertile male partner, lost to follow-up, biliopancreatic diversion patients who had not reached maximal weight loss	Intragastric balloon, adjustable gastric banding (LAGB), sleeve gastrectomy (LSG), gastric bypass (GB)	Mean 2.5 years	69/110 (62.7%) pregnant, all pregnancies ended with live births, no reported complications; Weight loss ≥ 5 BMI kg/m^2^ was a significant predictor of pregnancy (*p* < 0.001)
**Neto et al., 2012 [34]**	Retrospective	140	Morbidly obese patients undergoing Roux-en-Y gastric bypass (RYGB) with at least 5 years of follow-up	Lost to follow-up, mortality cases	Roux-en-Y gastric bypass (RYGB)	N/A	Infertility remission was sustained long-term, infertility not affected by weight regain
**Alatishe et al., 2013 [35]**	Retrospective	232	Women aged 18–45 who underwent bariatric surgery	N/A	Roux-en-Y gastric bypass (RYGB) (84.9%), laparoscopic adjustable gastric banding (LAGB) (8.2%), sleeve gastrectomy (3.4%)	Median 11 months (1.5–36 months)	24 live births (85.7%), 3 terminations (10.7%), 1 miscarriage (3.6%)
**Christofolini et al., 2014 [36]**	Retrospective	GI: 29 patients who had bariatric surgery; GII: 57 obese patients (BMI > 30 kg/m^2^); GIII: 94 patients	Women who underwent at least one cycle of controlled ovarian hyperstimulation (COH)	Previous weight reduction surgery for GII and GIII groups	Restrictive and/or malabsorptive bariatric surgery	Median 4.81 years (0.4–10 years)	No difference in pregnancy rates between post-bariatric surgery and control groups
**Eid et al., 2014 [37]**	Prospective	14	Premenopausal women aged 18–45 with PCOS diagnosis confirmed by an endocrinologist	Cushing syndrome, thyroid dysfunction, hyperprolactinemia, extreme androgen excess, pregnancy, immune diseases, or receiving other PCOS treatments	Laparoscopic Roux-en-Y gastric bypass (RYGB)	12 months	Not reported, but menstrual regularity restored in all patients by 6 months
**Goldman et al., 2016 [38]**	Retrospective	219	Women aged 18–45 who had a consultation for bariatric surgery	Patients who underwent sleeve gastrectomy or had an unidentified procedure	Roux-en-Y gastric bypass (RYGB) (111), Adjustable gastric banding (AGB) (66), controls (42)	<18 months	Lower odds of live birth post-AGB (OR = 0.19), increased miscarriage rate post-RYGB (OR = 9.81)
**Milone et al., 2017 [39]**	Retrospective	40	Obese women (BMI > 40 or BMI 35–39.9 with comorbidities) with previous ART failure	Male infertility, age > 38 years, AMH < 2 ng/mL, poor ovarian response, bariatric procedures other than sleeve gastrectomy	Sleeve gastrectomy	≤24 months	Pregnancy rate increased to 37.5% (15/40), live birth rate increased to 35% (14/40)
**Yau et al., 2017 [40]**	Retrospective	54	Women who became pregnant after bariatric surgery	15 pregnancies lost to follow-up, 4 ongoing pregnancies at data collection	Roux-en-Y gastric bypass (RYGB), sleeve gastrectomy (SG), laparoscopic adjustable gastric banding (LAGB)	<2 years (26 pregnancies), >2 years (15 pregnancies)	73 pregnancies, 96% healthy births in <2 years group, 93% in >2 years group; miscarriage rate higher in shorter interval group
**Nilsson-Condori et al., 2018 [41]**	Prospective	48	Women aged 18–35 with BMI 40.9 ± 3.6 kg/m^2^ undergoing RYGB	Cushing syndrome, thyroid dysfunction, hyperprolactinemia, extreme androgen excess	Laparoscopic Roux-en-Y gastric bypass (RYGB)	12 months	N/A
**Sahab Al kabbi et al., 2018 [42]**	Prospective	60	Women aged 18–40 years, BMI ≥ 36 kg/m^2^, without medical diseases or hormonal abnormalities, seeking bariatric surgery	Patients with PCOS, pregnancy, recent hormonal contraception use, endocrine disorders	Gastric sleeve (15), Gastric bypass (45)	65 ± 5 days	Improvement in menstrual cycle regularity post-op (from 58.3% to 86.7%)
**Vincentelli et al., 2018 [43]**	Retrospective	39	Women aged 18–45 years, BMI ≥ 40 or ≥35 kg/m^2^ with obesity-related comorbidities, failure of conservative treatment for at least 6–12 months	Women with ovarian surgery, postmenopausal women	Sleeve gastrectomy (23 women), Roux-en-Y gastric bypass (16 women)	12 months	AMH levels significantly decreased post-op
**Cruz et al., 2019 [44]**	Retrospective	42	Adult pregnant women who previously underwent RYGB, single-fetus pregnancy, gestational age ≤ 13 weeks, followed up by routine prenatal care	Other bariatric procedures, malabsorptive syndromes, neoplasias, liver/kidney diseases, failure to comply with vitamin supplementation, lack of prenatal follow-up	Roux-en-Y gastric bypass (RYGB)	Groups: ≤12 months (G1), >12 to <24 months (G2), ≥24 months (G3)	Higher neonatal complications and inadequate birth weight in G1, G3
**Gunakan et al., 2019 [45]**	Retrospective	23 (16 conceived ≤ 12 months, 7 conceived > 12 months post-surgery)	Women who conceived after laparoscopic sleeve gastrectomy	N/A	Laparoscopic sleeve gastrectomy	≤12 months (n = 16), >12 months (n = 7)	No significant differences in obstetric outcomes between early and late conception groups
**Menke et al., 2019 [46]**	Prospective	650	Women aged 18–44, no history of menopause, hysterectomy, or hormone replacement therapy	Women with missing reproductive history data	Various bariatric procedures, mostly Roux-en-Y gastric bypass (RYGB)	≤7 years	Higher conception rates in women with preoperative infertility, miscarriage rate 25%
**Grzegorczyk-Martin et al., 2020 [47]**	Retrospective	83 women with bariatric surgery vs. 166 BMI-matched controls vs. 83 non-operated severely obese women	Women undergoing their first IVF cycle, aged 18–43 years	Donor cycles, missing reproductive history data	Sleeve gastrectomy (60), adjustable gastric banding (13), Roux-en-Y gastric bypass (10)	Mean 2.98 ± 1.9 years before IVF	No significant difference in cumulative live birth rate (CLBR) between operated and non-operated BMI-matched women
**Jacamon et al., 2020 [48]**	Retrospective	52 women with bariatric surgery vs. 104 controls matched on pre-surgery BMI (Group A) vs. 104 controls matched on pre-pregnancy BMI (Group B)	Pregnant women with a history of bariatric surgery between April 2015 and January 2019	Twin pregnancies, pregnancies after gastric band removal, pregnancies occurring before bariatric surgery, refusal to participate, lack of matched control	Sleeve gastrectomy (58%), Roux-en-Y gastric bypass (37%), Adjustable gastric banding (5%)	Median 3 years, 35% of pregnancies occurred within 1-year post-op	71.2% pregnancies; Reduced gestational diabetes and large-for-gestational-age infants, but higher risk of small-for-gestational-age infants and neonatal intensive care unit admission
**Karadag et al., 2020 [49]**	Retrospective	144 pregnant women: 48 (Group A, <12 months post-surgery), 42 (Group B, >12 months post-surgery), 54 (Group C, obese controls)	Pregnant women who underwent laparoscopic sleeve gastrectomy (LSG)	Multiple pregnancies, miscarriages, intrauterine fetal demise	Laparoscopic sleeve gastrectomy (LSG)	7.8 ± 3.4 months (Group A), 25.8 ± 13.4 months (Group B)	Increased risk of small-for-gestational-age (SGA) infants in Group A, lower gestational diabetes mellitus (GDM) risk in both surgery groups vs. controls
**Ezzat et al., 2021 [50]**	Prospective	36	Obese women suffering from infertility due to PCOS (after excluding other causes), age 22–40 years, post-surgery BMI ≤ 35 kg/m^2^	Age > 40 years, post-surgery BMI > 35 kg/m^2^, medical disorders, other infertility factors, use of oral contraceptives	Laparoscopic sleeve gastrectomy (22 patients), laparoscopic gastric bypass (14 patients)	N/A	No pregnancies reported during the 1-year follow-up period
**Thornton et al., 2021 [51]**	Retrospective	460	Female patients aged 18 to 45 who underwent bariatric surgery from 2013 to 2018	History of permanent contraception, hysterectomy, tubal ligation	N/A	18 months	6% pregnancy rate within 18 months post-surgery
**Snoek et al., 2024 [52]**	Prospective	97 post-gastric bypass (pGB) pregnancies vs. 440 non-bariatric pregnancies	Singleton pregnancies after gastric bypass surgery with available fetal growth and birthweight data	Multiple pregnancies, pregnancies with missing growth data	Roux-en-Y gastric bypass (RYGB)	Median 18.3 months (IQR 9.4–28.5)	Increased risk of small-for-gestational-age (SGA) infants, reduced birthweight, but no significant impact on maternal pregnancy complications
**Joly et al., 2024 [53]**	Retrospective	244	Patients with a history of sleeve or bypass who delivered between 2004 and 2021 after their first pregnancy post-bariatric surgery	Subsequent pregnancies post-bariatric surgery, gastric band procedures, deliveries in another center, multiple pregnancies, terminations of pregnancy, miscarriages	Sleeve gastrectomy (145), Roux-en-Y gastric bypass (99)	33.7 months (sleeve), 40.9 months (bypass)	Higher incidence of SGA (26.2% sleeve vs. 22.22% bypass), lower preterm birth in sleeve (3.45%) vs. bypass (12.12%)
**Samarasinghe et al., 2024 [54]**	RCT	80 (40 bariatric surgery, 40 medical care)	- **≥18 years**. - **PCOS** - **BMI ≥ 35 kg/m**^2^. - Presence of **oligomenorrhoea or amenorrhea**, defined as: cycle length **< 21 days or >35 days, <8 cycles in the past 12 months** or **absence of menstruation**	- **Diabetes** - Inability of **effective non-hormonal contraception** - **Current pregnancy or breastfeeding** - **Gastro-esophageal reflux disease** - **Avoid conception 12–18 months post-bariatric surgery**	Vertical sleeve gastrectomy (VSG)	12–18 months post-bariatric surgery	2.5 times more spontaneous ovulations 93% women with menstrual dysfunction pre-op complete resolution within 6 months post-op 1 pregnancy in the surgical group 2 pregnancies in the medical group

This table summarizes the methodological characteristics of the studies included in this systematic review, including the study design, sample size, inclusion and exclusion criteria, type of bariatric surgery performed, time interval between bariatric surgery and pregnancy, and important pregnancy outcomes. The table presents a systematic comparison of the effects of various bariatric surgeries on fertility and pregnancy outcomes, such as improved menstrual control, ovulatory function, conception rates, and pregnancy problems.

**Table 3:** Patient characteristics.

Author	Number of Patients	Age	BMI Before Surgery	BMI After Surgery	Follow-Up Time	PCOS (Number of Patients)	Cause of Infertility
**Deitel et al., 1988 [22]**	138	34.8 ± 8.7 (17–56)	45.5 ± 6.3	29.1 ± 4.7	2–5 years	N/A	Menstrual irregularities (40.4%) Hyperandrogenism (31.9%) Obesity-related endocrine dysfunction Stress urinary incontinence and pelvic floor dysfunction (61.2%)
**Bilenka et al., 1995 [23]**	9	24 to 36 years (mean 31.9)	35–50 kg/m^2^.	27–38 kg/m^2^	N/A	N/A	Morbid obesity
**Sheiner et al., 2004 [24]**	298	29.1 ± 5.7	N/A	10.7% remained obese (BMI ≥ 30)	N/A	N/A	Obesity
**Marceau et al., 2004 [25]**	132	Mean 37.3 ± 10.3 years at surgery	Mean 47.1 ± 8.3	Mean 30.9 ± 6.4	Mean 7.9 ± 4.2 years (range 2–18 years)	N/A	Obesity-related anovulation
**Eid et al., 2005 [37]**	24	34 ± 9.7 years	50 ± 7.5	30 ± 4.5	27.5 ± 16 months	24	PCOS-related anovulation
**Rochester et al., 2009 [27]**	9	38.0 ± 8.0 years	47.3 ± 5.2 kg/m^2^	32.0 ± 2.9 kg/m^2^	6–12 months post-op	N/A	Obesity
**Gosman et al., 2010 [28]**	1538	44.8 ± 11.2 years (18–78)	47.2 ± 7.5 kg/m^2^ (33.8–87.3)	N/A	N/A	201 (13.1%)	Obesity
**Sheiner et al., 2011 [29]**	489	30.5 ± 4.7 years (<12 months), 31.3 ± 5.1 years (>12 months)	42.2 ± 5.1 kg/m^2^ (<12 months), 42.6 ± 5.0 kg/m^2^ (>12 months)	N/A	N/A	N/A	N/A
**Musella et al., 2011 [30]**	19	31 ± 4.8 years (22–39)	41 ± 2.7 kg/m^2^ (36–45)	Decreased by 7.5 ± 1.1 BMI units in 18 patients	At least 1 year	N/A	Obesity
**Facchiano et al., 2012 [31]**	36	30.4 ± 4.7 years (LAGB), 31.2 ± 3.9 years (LRYGB)	42.7 ± 3.8 kg/m^2^ (LAGB), 50.5 ± 4.9 kg/m^2^ (LRYGB)	N/A	N/A	N/A	Obesity
**Jamal et al., 2012 [32]**	20	32 ± 5.8 years (22–42)	52.8 ± 9.08 kg/m^2^ (37–76)	34.3 ± 5.7 kg/m^2^	Mean 46.7 months (15–123 months)	20	PCOS-related anovulation, menstrual irregularity, hyperandrogenism
**Legro et al., 2012 [33]**	29	34.5 ± 4.3 years	49 ± 7 kg/m^2^	34.3 ± 5.7 kg/m^2^ at 12 months	Up to 24 months	N/A	Obesity, ovulatory irregularities
**Musella et al., 2012 [55]**	110	29.3 ± 3.9 years (pregnant group), 28.6 ± 3.2 years (non-pregnant group)	43.9 ± 4.1 kg/m^2^ (pregnant group), 45.1 ± 3.7 kg/m^2^ (non-pregnant group)	34.2 ± 2.4 kg/m^2^ (pregnant group), 41.5 ± 2.8 kg/m^2^ (non-pregnant group)	>2.5 years	N/A	Obesity
**Neto et al., 2012 [34]**	140	41.4 ± 10.6 years (range 19–62)	52.5 ± 7.9 kg/m^2^	29.3 ± 5.2 kg/m^2^ (nadir), 33.7 ± 6.3 kg/m^2^ (final follow-up)	Mean 90 months (60–155 months)	N/A	Amenorrhea, irregular menstrual cycles, inability to conceive after 6 months
**Alatishe et al., 2013 [35]**	232	34.0 ± 5.9 years	50.6 ± 7.2 kg/m^2^	N/A	Median 30 months	N/A	Obesity
**Christofolini et al., 2014 [36]**	GI: 29 patients undergone bariatric surgery; GII: 57 obese patients (BMI > 30 kg/m^2^); GIII: 94 patients	35.0 ± 3.6 years	N/A	26.6 (22.8–35.8) kg/m^2^	N/A	1/29	Idiopathic (24.1%), male factor (34.4%), endometriosis (13.7%), tubal factor (20.6%)
**Eid et al., 2014 [37]**	14	36.3 ± 8.4 years (range 19–49)	44.8 ± 5.9 kg/m^2^ (range 37.0–56.6)	29.2 ± 5.9 kg/m^2^ at 12 months	12 months	14	PCOS-related anovulation, menstrual irregularities, and hyperandrogenism
**Goldman et al., 2016 [38]**	219	Mean 39.4 years (RYGB), 40.4 years (AGB)	N/A	Decrease in BMI: 14.71 ± 6.35 (RYGB), 9.17 ± 6.14 (AGB)	<18 months	28 (25.5%) in RYGB group, 16 (24.6%) in AGB group	PCOS, obesity
**Milone et al., 2017 [39]**	40	31.4 ± 4.7 years (pre-surgery), 32.4 ± 4.4 years (post-surgery)	40.7 ± 2 kg/m^2^	35 ± 2.6 kg/m^2^	≤24 months	N/A	Idiopathic infertility
**Yau et al., 2017 [40]**	54	32.1 years (<2 years), 31.1 years (>2 years), *p* = 0.539	46.7 kg/m^2^ (<2 years), 44.5 kg/m^2^ (>2 years), *p* = 0.366	29.8 kg/m^2^ (<2 years), 32.6 kg/m^2^ (>2 years), *p* = 0.205	N/A	N/A	N/A
**Nilsson-Condori et al., 2018 [41]**	48	26.5 ± 4.3 years	40.9 ± 3.6 kg/m^2^	25.4 ± 6.4 kg/m^2^ at 12 months	12 months	10	N/A
**Sahab Al kabbi et al., 2018 [42]**	60	32.77 ± 6.64 years (18–40)	48.95 ± 7.46 kg/m^2^	41.18 ± 6.76 kg/m^2^	Mean 65 ± 5 days	Excluded from study	N/A
**Vincentelli et al., 2018 [43]**	39	34.6 ± 1.1 years	45.4 ± 1.0 kg/m^2^	31.4 ± 0.9 kg/m^2^ at 12 months	12 months	6 (15%)	PCOS, obesity, ART failures in 4 patients (endometriosis, impaired ovarian reserve, male factor infertility)
**Cruz et al., 2019 [44]**	42	22–39 years	46.35 ± 7.51 kg/m^2^ (G1), 44.62 ± 6.64 kg/m^2^ (G2), 40.83 ± 3.05 kg/m^2^ (G3)	27.80 ± 3.98 kg/m^2^ (G1), 27.74 ± 3.34 kg/m^2^ (G2), 25.73 ± 2.58 kg/m^2^ (G3)	N/A	N/A	N/A
**Gunakan et al., 2019 [45]**	23	32.4 ± 4.2 years	46.6 ± 4.4 kg/m^2^	29.7 ± 3.8 kg/m^2^	N/A	N/A	9 patients (39.1%) history of infertility
**Menke et al., 2019 [46]**	650	Median 34 years (30–39)	N/A	N/A	<7 years	N/A	PCOS, obesity-related infertility, prior ART failure
**Grzegorczyk-Martin et al., 2020 [47]**	83	33.1 ± 4.4 years	43.6 kg/m^2^ (39–54)	28.9 ± 4.7 kg/m^2^	Mean 2.98 years before IVF	25.3%	Ovulatory (16.7%), tubal (15.4%), male (32.1%), idiopathic (16.7%), endometriosis (2.6%), mixed (16.7%)
**Jacamon et al., 2020 [48]**	52	31.1 ± 5.0 years	46.0 ± 4.6 kg/m^2^	29.4 ± 6.1 kg/m^2^	<3 years	N/A	N/A
**Karadag et al., 2020 [49]**	144	30.3 ± 5.1 years (Group A), 28.8 ± 4.7 years (Group B), 27.5 ± 3.9 years (Group C)	44.37 ± 3.66 kg/m^2^ (Group A), 42.71 ± 3.71 kg/m^2^ (Group B)	32.83 ± 3.63 kg/m^2^ (Group A), 28.90 ± 2.84 kg/m^2^ (Group B)	<25.8 months	N/A	N/A
**Ezzat et al., 2021 [50]**	36	27.2 ± 4.2 years (21–36)	43.6 ± 1.76 kg/m^2^	33.5 ± 1.36 kg/m^2^ (6 months), 29.1 ± 1.17 kg/m^2^ (1 year)	1 year	36	PCOS
**Thornton et al., 2021 [51]**	460	N/A	N/A	N/A	18 months	N/A	N/A
**Snoek et al., 2024 [52]**	97	Median 29.2 years (IQR 26.0–32.2)	Median 43.6 kg/m^2^	Median 29.8 kg/m^2^ at conception	N/A	N/A	N/A
**Joly et al., 2024 [53]**	244	32.1 years (sleeve), 32.3 years (bypass)	43.5 kg/m^2^ (sleeve), 46.5 kg/m^2^ (bypass)	30.4 kg/m^2^ (sleeve), 31.1 kg/m^2^ (bypass)	N/A	N/A	N/A
**Samarasinghe et al., 2024 [54]**	80 (40 bariatric surgery, 40 medical care)	Mean 32 ± 6 years (median 31 y.o)	- **Surgical group: 45.0 kg/m**^2^ **(41.4–50.8** kg/m^2^) - **Medical group: 41.9 kg/m**^2^ (**38.1–45.1** kg/m^2^)	- **Surgical group: 32.8 kg/m**^2^ (**29.9–37.1** kg/m^2^) - **Medical group: 41.6 kg/m**^2^ (**37.7–44.9** kg/m^2^)	52 weeks (1 year)	80	PCOS (79% oligomenorrhoea or amenorrhea) Obesity

**Table 4:** Fertility, endocrine outcomes, and vitamin deficiencies post-bariatric surgery.

Author	Type of Bariatric Surgery	Fertility	Vitamin Status Post-op	Endocrine Changes (AMH, LH, FSH, Estradiol, Testosterone, Androstenedione)	Miscarriage Rate
**Deitel et al., 1988 [22]**	Various procedures	Improved	N/A	N/A	N/A
**Bilenka et al., 1995 [23]**	Vertical banded gastroplasty (VBG)	Improved	N/A	N/A	7.1% after VBG vs. 38.9% before
**Sheiner et al., 2004 [24]**	Restrictive and malabsorptive (open/laparoscopic)	N/A	Risk of anemia (iron, folate, B12 deficiency)	N/A	N/A
**Marceau et al., 2004 [25]**	Biliopancreatic diversion (BPD)	Improved	Serum albumin decreased from 40.4 ± 2.7 to 35.7 ± 5.5 g/L; 4 cases of severe hypoalbuminemia (<26 g/L) requiring parenteral nutrition	N/A	Pre-op: 21.6% (341/1577 pregnancies); post-op: 26% (57/219 pregnancies)
**Eid et al., 2005 [26]**	Laparoscopic Roux-en-Y gastric bypass	Improved	N/A	N/A	N/A
**Rochester et al., 2009 [27]**	Gastric bypass (10), Lap-band (6)	Improved	N/A	LH increased, FSH stable, estradiol decreased, progesterone increased but lower than controls	N/A
**Gosman et al., 2010 [28]**	Gastric bypass (74.1%), adjustable gastric banding (23.2%)	N/A	N/A	N/A	25.2% had at least one miscarriage, miscarriage in 17.4% of pregnancies
**Sheiner et al., 2011 [29]**	LAGB (61.5% < 12 months, 41.0% > 12 months), SRVG, VBG, RGB	N/A	N/A	N/A	N/A
**Musella et al., 2011 [55]**	Intragastric balloon (BIB^®^ Bioenterics Intragastric Balloon)	Improved	N/A	N/A	None
**Facchiano et al., 2012 [31]**	LAGB and LRYGB	Improved; no significant difference between LAGB and LRYGB	N/A	N/A	2 miscarriages (LRYGB group) at 16 and 22 weeks
**Jamal et al., 2012 [32]**	Roux-en-Y gastric bypass (RYGB)	Improved	N/A	Menstrual cycle improved (82%), hirsutism resolved (29%), T2DM resolved (77.8%)	N/A
**Legro et al., 2012 [33]**	Roux-en-Y gastric bypass (RYGB)	Not improved	N/A	Increased SHBG, decreased testosterone and estradiol, shortened follicular phase	N/A
**Musella et al., 2012 [55]**	Intragastric balloon, LAGB, LSG, GB	Improved	N/A	N/A	None
**Neto et al., 2012 [34]**	Roux-en-Y gastric bypass (RYGB)	Not improved	N/A	N/A	N/A
**Alatishe et al., 2013 [35]**	Roux-en-Y gastric bypass (RYGB), LAGB, sleeve gastrectomy	Improved	N/A	N/A	Pre-surgery: 20.7%; post-surgery: 3.6%
**Christofolini et al., 2014 [36]**	Restrictive and/or malabsorptive bariatric surgery	Not improved	N/A	N/A	N/A
**Eid et al., 2014 [37]**	Laparoscopic Roux-en-Y gastric bypass (RYGB)	Menstrual regularity restored in all patients by 6 months	N/A	Testosterone decreased, fasting insulin decreased, LH increased, FSH stable	N/A
**Goldman et al., 2016 [38]**	Roux-en-Y gastric bypass (RYGB), adjustable gastric banding (AGB)	Menstrual cycle regularity improved post-RYGB (OR = 0.21), no significant improvement post-AGB	N/A	N/A	Higher post-RYGB compared to controls (OR = 9.81)
**Milone et al., 2017 [39]**	Sleeve gastrectomy	Improved	N/A	FSH stable, AMH stable	2.5% (1/40)
**Yau et al., 2017 [40]**	Roux-en-Y gastric bypass (RYGB), sleeve gastrectomy (SG), laparoscopic adjustable gastric banding (LAGB)	Improved	Vitamin B1 deficiency: 46% (<2 years), 20% (>2 years), *p* = 0.177; vitamin D deficiency: 65% (<2 years), 87% (>2 years), *p* = 0.168	N/A	Higher in <2 years group (7/33, 21%) vs. >2 years group (1/16, 6%)
**Nilsson-Condori et al., 2018 [41]**	Laparoscopic Roux-en-Y gastric bypass (RYGB)	N/A	N/A	AMH decreased, FAI decreased, SHBG increased, testosterone decreased, estradiol increased	N/A
**Sahab Al kabbi et al., 2018 [42]**	Gastric sleeve, gastric bypass	N/A	N/A	AMH decreased significantly post-op	N/A
**Vincentelli et al., 2018 [43]**	Sleeve gastrectomy, Roux-en-Y gastric bypass	N/A	N/A	AMH decreased significantly	N/A
**Cruz et al., 2019 [44]**	Roux-en-Y gastric bypass (RYGB)	N/A	N/A	N/A	23.8% (G1), 47.6% (G2), 28.6% (G3)
**Gunakan et al., 2019 [45]**	Laparoscopic sleeve gastrectomy	Improved	N/A	N/A	3 cases (13%)
**Menke et al., 2019 [46]**	Various procedures, predominantly RYGB	Improved	N/A	N/A	25%
**Grzegorczyk-Martin et al., 2020 [47]**	Sleeve gastrectomy, adjustable gastric banding, Roux-en-Y gastric bypass	Stable	N/A	N/A	38.7% (operated group), 35.8% (BMI-matched non-operated), 56.5% (severely obese non-operated)
**Jacamon et al., 2020 [48]**	Sleeve gastrectomy, Roux-en-Y gastric bypass, adjustable gastric banding	Improved	N/A	N/A	29% post-surgery, compared to 32% (Group A) and 22% (Group B)
**Karadag et al., 2020 [49]**	Laparoscopic sleeve gastrectomy (LSG)	N/A	N/A	N/A	N/A
**Ezzat et al., 2021 [50]**	Laparoscopic sleeve gastrectomy, laparoscopic gastric bypass	N/A	N/A	Free and total testosterone decreased, SHBG increased, Free androgen index decreased	N/A
**Thornton et al., 2021 [51]**	N/A	Improved	N/A	N/A	N/A
**Snoek et al., 2024 [52]**	Roux-en-Y gastric bypass (RYGB)	N/A	Lower vitamin serum levels post-op	N/A	N/A
**Joly et al., 2024 [53]**	Sleeve gastrectomy, Roux-en-Y gastric bypass	N/A	Higher vitamin deficiencies in bypass group	N/A	N/A
**Samarasinghe et al., 2024 [54]**	Vertical sleeve gastrectomy (VSG)	Improved	**- Iron deficiency: 3 patients (8%).** **- Vitamin B12 deficiency: 0 patients (0%)** **- Folic acid deficiency: 7 patients (19%)** **- Vitamin D deficiency: 5 patients (14%)**	AMH decreased in the surgical group - **Baseline: 46.4 pmol/L** (medical) vs. **31.2 pmol/L** (surgical). - **52 weeks: 40.5 pmol/L** (medical) vs. **23.7 pmol/L** (surgical) (***p* = 0.0061**). LH non-significant decrease - **Baseline: 8.3 U/L** (medical) vs. **9.2 U/L** (surgical) - **52 weeks: 8.7 U/L** (medical) vs. **6.4 U/L** (surgical) (***p* = 0.080**) FSH stable - **Baseline: 5.0 U/L** (medical) vs. **5.3 U/L** (surgical) - **52 weeks: 5.5 U/L** (medical) vs. **5.3 U/L** (surgical) (***p* = 0.65**) E2 no significant difference - **Baseline: 267.4 pmol/L** (medical) vs. **356.7 pmol/L** (surgical) - **52 weeks: 290.3 pmol/L** (medical) vs. **303.8 pmol/L** (surgical) (***p* = 0.82**) Testosterone **significant decrease** - **Baseline: 2.0 nmol/L** (medical) vs. **1.8 nmol/L** (surgical) - **52 weeks: 1.8 nmol/L** (medical) vs. **1.2 nmol/L** (surgical) (***p* = 0.0065**) Androstenedione **significant decrease** - **Baseline: 7.0 nmol/L** (medical) vs. **6.4 nmol/L** (surgical) - **52 weeks: 5.7 nmol/L** (medical) vs. **4.5 nmol/L** (surgical) (***p* = 0.039**)	N/A

Table 4 summarizes the effects of various bariatric surgery types on fertility, endocrine markers, vitamin status, and miscarriage rates. It discusses variations in reproductive hormone levels (AMH, LH, FSH, estradiol, testosterone, and androstenedione), menstrual cycle regularity, and post-operative problems such as vitamin shortages. Furthermore, it examines the effect of bariatric surgery on miscarriage rates in post-surgical pregnancies, allowing for an assessment of the possible risks and advantages of various surgical methods.

**Table 5:** Pregnancy complications.

Author	IUGR	GDM	Preeclampsia	Congenital Malformation	Other Complications
**Deitel et al., 1988 [22]**	Decreased	Decreased	Decreased	N/A	Decreased
**Bilenka et al., 1995 [23]**	None	Decreased	Decreased	None	Decreased
**Sheiner et al., 2004 [24]**	5%	9.4%	5.7%	5%	Higher rates of previous CS, labor induction, PROM
**Marceau et al., 2004 [25]**	Increased Pre-op 3.1% (20/638) Post-op: 9.6% (15/156)	N/A	Increased Pre-op 0 Post-op 1	Increased Pre-op 2.6% (33/1245) Post-op 4.2% (7/166)	Pre-op miscarriage rate: 21.6% (341/1577); cesarean section rate: not reported Post-op: miscarriage rate: 26% (57/219 non-terminated pregnancies); cesarean section rate: 20.3%; 4 severe hypoalbuminemia requiring parenteral nutrition; 1 placental detachment; 1 intestinal obstruction
**Eid et al., 2005 [26]**	N/A	N/A	N/A	N/A	N/A
**Rochester et al., 2009 [27]**	N/A	N/A	N/A	N/A	N/A
**Gosman et al., 2010 [28]**	N/A	N/A	N/A	N/A	Higher stillbirth rate (13.2 per 1000 live births) compared to U.S. general population (6.2 per 1000 live births)
**Sheiner et al., 2011 [29]**	3.8% (<12 months), 2.3% (>12 months), *p* = 0.396	10.5% (<12 months), 7.3% (>12 months), *p* = 0.159	15.4% (<12 months), 11.2% (>12 months), *p* = 0.392	1.9% (<12 months), 1.3% (>12 months), *p* = 0.458	Higher rate of LAGB in early group (61.5%) vs. late group (41.0%)
**Musella et al., 2011 [30]**	None	None	None	None	None
**Facchiano et al., 2012 [31]**	1 case (LAGB), 2 cases (LRYGB)	3 cases (LAGB), 2 cases (LRYGB)	3 cases (LAGB), 0 cases (LRYGB)	None	7 cesarean deliveries (LAGB), 8 cesarean deliveries (LRYGB); 2 postpartum hemorrhages (LAGB); minor complications secondary to bariatric surgery in LRYGB group
**Jamal et al., 2012 [32]**	N/A	N/A	N/A	N/A	N/A
**Legro et al., 2012 [33]**	N/A	N/A	N/A	N/A	N/A
**Musella et al., 2012 [55]**	N/A	N/A	N/A	N/A	N/A
**Neto et al., 2012 [34]**	N/A	N/A	N/A	N/A	N/A
**Alatishe et al., 2013 [35]**	N/A	N/A	N/A	N/A	None
**Christofolini et al., 2014 [36]**	N/A	N/A	N/A	N/A	N/A
**Eid et al., 2014 [37]**	N/A	N/A	N/A	N/A	N/A
**Goldman et al., 2016 [38]**	N/A	N/A	N/A	N/A	Lower neonatal birth weight post-RYGB (2983 g vs. 3583 g in controls)
**Milone et al., 2017 [39]**	N/A	N/A	N/A	N/A	N/A
**Yau et al., 2017 [40]**	N/A	0% (<2 years), 6% (>2 years), *p* = 0.984	4% (<2 years), 6% (>2 years), *p* = 0.995	N/A	Gestational hypertension: 12% in both groups, hyperemesis gravidarum: 31% (<2 years), 40% (>2 years)
**Nilsson-Condori et al., 2018 [41]**	N/A	N/A	N/A	N/A	N/A
**Sahab Al kabbi et al., 2018 [42]**	N/A	N/A	N/A	N/A	N/A
**Vincentelli et al., 2018 [43]**	N/A	N/A	N/A	N/A	N/A
**Cruz et al., 2019 [44]**	N/A	N/A	N/A	N/A	Neonatal complications: 30% (G1), 5% (G2), 0% (G3); Weight inadequacy at birth: 20% (G1), 0% (G2, G3)
**Gunakan et al., 2019 [45]**	2 cases (10%)	2 cases (10%)	2 cases (10%)	Not reported	Preterm delivery in 1 case (5%), no significant difference between groups
**Menke et al., 2019 [46]**	N/A	N/A	N/A	N/A	N/A
**Grzegorczyk-Martin et al., 2020 [47]**	N/A	N/A	N/A	N/A	N/A
**Jacamon et al., 2020 [48]**	N/A	Reduced from 44% (controls) to 12% (post-bariatric surgery)	Reduced from 13% (controls) to 2% (post-bariatric surgery)	N/A	Higher risk of small-for-gestational-age infants and NICU admission in post-bariatric surgery group
**Karadag et al., 2020 [49]**	N/A	Lower in surgery groups vs. control (4.3% Group A, 9.5% Group B, 29.6% Group C, *p* = 0.004)	8.3% (Group A), 9.5% (Group B), 7.4% (Group C), *p* = 0.695	N/A	SGA risk higher in Group A (22.9%) vs. Group B (11.9%) and Group C (7.4%), *p* = 0.025
**Ezzat et al., 2021 [50]**	N/A	N/A	N/A	N/A	N/A
**Thornton et al., 2021 [51]**	N/A	N/A	N/A	N/A	N/A
**Snoek et al., 2024 [52]**	N/A	No significant difference	No significant difference	N/A	Significantly higher risk of small-for-gestational-age (SGA) infants
**Joly et al., 2024 [53]**	N/A	No significant difference	No significant difference	N/A	Higher risk of SGA in sleeve (26.2%) vs. bypass (22.22%), higher preterm birth in bypass group (12.12%) vs. sleeve (3.45%)
**Samarasinghe et al., 2024 [54]**	N/A	N/A	N/A	N/A	N/A

## Supplementary Materials

The following supporting information can be downloaded at: https://www.mdpi.com/article/10.3390/life15050758/s1, Supplementary Table S1. Full Search Strategies Used for Each Database.
