# Supplementary material for: The Relationship Between Obesity, Bariatric Surgery, and Infertility: A Systematic Review
Source: Life (Basel). 2025 May 9;15(5):758. doi: 10.3390/life15050758 (PMC12113062; doi:10.3390/life15050758)
Supplement: Supplementary file 1 [file life-15-00758-s001.zip › life-3566987-supplementary.pdf]

Supplementary Table S1. Full Search Strategies Used for Each Database

Database	Search Strategy Used	Date of Search	Language Restrictions
PubMed	("bariatric surgery"[MeSH Terms] OR "bariatric surgery"[All Fields] OR "weight loss surgery") AND ("fertility"[MeSH Terms] OR "infertility"[All Fields] OR "reproductive health" OR "PCOS" OR "ovulation" OR "pregnancy")	October 2023	English only
Scopus	TITLE-ABS-KEY ("bariatric surgery" OR "weight loss surgery") AND TITLE-ABS-KEY ("fertility" OR "infertility" OR "pregnancy" OR "PCOS" OR "ovulation")	October 2023	English only
Embase	('bariatric surgery'/exp OR 'bariatric surgery' OR 'weight loss surgery') AND ('fertility'/exp OR 'infertility' OR 'pregnancy' OR 'polycystic ovary syndrome' OR 'reproductive function')	October 2023	English only
Google Scholar	Allintitle: "bariatric surgery" AND ("fertility" OR "pregnancy" OR "infertility" OR "PCOS" OR "reproduction")	October 2023	English only
